# Macronutrient deficiency reduces growth and influences vegetation indices of greenhouse grown ornamental and vegetable plants as measured by the TraitFinder digital phenotyping system

**DOI:** 10.3389/fpls.2026.1702429

**Published:** 2026-05-12

**Authors:** Juan Quijia-Pillajo, Janhavi Maurya, Laura J. Chapin, Michelle L. Jones

**Affiliations:** Department of Horticulture and Crop Science, The Ohio State University, Wooster, OH, United States

**Keywords:** celosia, coleus, marigold, nutrient stress, petunia, PlantEye F600, soilless substrate, tomato

## Abstract

**Introduction:**

Recent technological advances in high resolution image capture and analysis have led to increased adoption of high-throughput digital phenotyping in plant science research. High-throughput digital phenotyping provides a nondestructive method to quantify changes in plant growth and health in response to environmental factors or developmental cues. Moreover, it allows researchers to conduct large experiments in a time- and cost-efficient manner. The TraitFinder is a digital phenotyping system developed by Phenospex (Heerlen, Netherlands) that measures plant morphological (e.g., digital biomass) and spectral (leaf light reflectance) information. Leaf reflectance is presented as five vegetation indices (e.g., normalized difference vegetation index).

**Methods:**

This project evaluated nitrogen (N), phosphorus (P), and potassium (K) deficiency in greenhouse grown ornamental and vegetable plants using the TraitFinder. Plant species included celosia, coleus, marigold, petunia, and tomato. Plants were fertilized with a complete Hoagland’s solution (control), and three modified solutions: Hoagland’s solution without nitrogen (-N), phosphorus (-P), or potassium (-K). Each plant species was evaluated separately with eight replicate plants per treatment, organized as a randomized complete block design.

**Results:**

Treatment with -N, -P, and -K solutions resulted in reduced vegetative growth and decreased concentration of the corresponding macronutrient in leaf tissue for all species evaluated. We observed that the presence of flowers would negatively affect calculations of the vegetation indices due to their distinct spectral properties; therefore, flowers must be excluded to accurately quantify plant health parameters. In general, we observed a common trend where GLI (green leaf index) and NDVI (normalized difference vegetation index) decreased, and NPCI (normalized pigment chlorophyll index) and PSRI (plant senescence reflectance index) increased in response to macronutrient deficiency. The measure of GLI, NDVI, NPCI, and PSRI were different from the control plants, but these observations were dependent on the nutrient deficiency and species tested.

**Discussion:**

Our results underscore the importance of accounting for species-specific spectral signatures when assessing plant responses to nutrient deficiencies. This project also provides reference values for interpreting vegetation indices, offering valuable guidance for scientists implementing digital phenotyping in their experimental protocols. Digital phenotyping can significantly improve experimental throughput and provide quantitative insights into plant health.

## 1 Introduction

High-throughput digital phenotyping technologies are quickly being adopted by plant researchers. Compared to low-throughput phenotyping (manual measurements), high-throughput phenotyping allows for non-destructive evaluation of large experiments (high number of replicates or treatments) in a time- and cost-efficient manner. The TraitFinder is a digital phenotyping system developed by Phenospex (Heerlen, Netherlands) that uses two PlantEye F600 multispectral 3D laser scanners to quantify plant phenotypes. The PlantEye F600 scanners create a 3D representation of the plants and capture multispectral information. Several morphological parameters are calculated from the 3D representation, including digital biomass and leaf area. Digital growth measurements show high correlation with manual measurements such as growth index and destructive dry weight measurements (Manavalan et al., 2021; Bazhenov et al., 2023; Quijia Pillajo et al., 2023). The multispectral information consists of the amount of light reflected in the green (G), blue (B), red (R), and near-infrared (NIR) wavelengths, and it is used to calculate vegetation indices used for plant health assessment. Recent research projects conducted with the TraitFinder have used plant models like amaranth (Nyonje et al., 2021), basil (Lazarević et al., 2021), soybean (Manavalan et al., 2021), common bean (Alghafri, 2022), wheat, and rye (Bazhenov et al., 2023). Bazhenov et al. (2023) quantified growth and health in wheat and rye plants fertilized with rock phosphate, and Manavalan et al. (2021) conducted a high-throughput screen of biological seed treatments in soybean. We have also recently taken advantage of the TraitFinder system to phenotype petunia and marigold treated with beneficial microorganisms (Quijia Pillajo et al., 2023; Quijia-Pillajo et al., 2025), but we have not found other reports working with ornamental crops.

Leaf biochemical and biophysical characteristics influence the absorption and reflection of light across different wavelengths of the solar spectrum. Variations in reflectance at specific wavelengths provide valuable insights into canopy health (Jakomulska et al., 2002). Leaf pigment content (i.e., chlorophylls, anthocyanins, and carotenoids) and leaf structure influence reflectance in the visible (RGB) and near-infrared (NIR) region of the light spectrum, respectively (Slaton et al., 2001; Sims and Gamon, 2002; Ollinger, 2011). Green and healthy foliage commonly shows high NIR reflectance and low RGB reflectance (high RGB absorbance) (Jakomulska et al., 2002). The reflected light can be measured at each wavelength by multispectral and hyperspectral cameras (Jangra et al., 2021). The spectral reflectance information measured at two or more wavelengths is used to calculate vegetation indices, which are widely used to quantitatively evaluate plants under stress (Montero et al., 2023). The TraitFinder system provides four vegetation indices: the green leaf index (GLI), normalized difference vegetation index (NDVI), normalized pigment chlorophyll index (NPCI), and plant senescence reflectance index (PSRI). Spectral data is also used to calculate canopy’s hue, saturation, and lightness. These parameters provide detailed analysis of plant color change in response to a treatment (Nguyen et al., 2021). For greenhouse ornamentals, foliage and flower colors are indicators of quality that can be influenced by nutrient deficiencies (Nau et al., 2021).

GLI and NDVI are commonly used to measure green vegetation and greenness. GLI is calculated using the spectral reflectance at the green (G), red (R), and blue (B) wavelengths [GLI = (2 × G – R – B)/(2 × G + R + B)] (Louhaichi et al., 2001). NDVI is calculated using the spectral reflectance at the red (R) and near-infrared (NIR) wavelengths [NDVI = (NIR – R)/(NIR + R)]. In general, GLI and NDVI values decrease under nutrient limitation (Farias et al., 2023). NDVI has been used as an estimator of yield (Jasim et al., 2020), nutrient uptake (N, P, and K) (Farias et al., 2023), and heat stress (Tafesse et al., 2022). Healthy vegetation commonly has a GLI and NDVI above 0.2 and 0.5, respectively (Jakomulska et al., 2002; Moritz et al., 2024).

NPCI and PSRI are used to quantify plant stress responses and senescence. NPCI is calculated using the spectral reflectance at the red (R) and blue (B) wavelengths [NPCI = (R − B)/(R + B)]. PSRI is calculated using the spectral reflectance at the blue (B), red (R), and near-infrared NIR wavelengths [PSRI = (R − B)/NIR]. NPCI is an indicator of N deficiency and abiotic stress (i.e., water and heat) that negatively correlates with leaf chlorophyll content (Peñuelas et al., 1994; Tafesse et al., 2022). PSRI is used to quantify leaf senescence or fruit ripening. Senescence induced chlorophyll degradation is accompanied by an increase in the reflectance between 550 nm and 740 nm (Merzlyak et al., 1999). PSRI and NPCI correlate with the leaf carotenoid/chlorophyll content ratio (Peñuelas et al., 1994; Merzlyak et al., 1999; Sims and Gamon, 2002). The NPCI and PSRI of healthy vegetation ranges from −0.1 to 0.2 and −0.1 to 0, respectively (Bazhenov et al., 2023). NPCI and PSRI values increase in response to abiotic stress (i.e., nutrient limitation and heat) (Peñuelas et al., 1994; Merzlyak et al., 1999; Tafesse et al., 2022).

The leaf spectral reflectance profile can be influenced by many variables including plant species, phenological stage, and health status (Sims and Gamon, 2002; Ollinger, 2011; Shi et al., 2012). For example, Sims and Gamon (2002) showed that the correlation between SR_705_ (R_750_/R_705,_ known as the simple ratio) and chlorophyll content is influenced by leaf structure. Senescence driven pigment degradation is species dependent and also influences PSRI values (Merzlyak et al., 1999). Preliminary tests are required to understand the spectral signatures of plant models under normal and experimental conditions (Shi et al., 2012; Jasim et al., 2020). Understanding the optimal range for the vegetation indices is necessary to advance the usage of these systems in plant research. However, there are few reports about reference values of GLI, NDVI, NPCI, and PSRI corresponding to healthy or nutrient stressed ornamental crops. Quijia Pillajo et al. (2023) reported an NDVI of about 0.7 in petunias grown under low fertility (50 mg L^-1^ N from 15N-2.2P-12.5K-2.9Ca-1.2Mg), but not under optimal fertility. NDVI correlates with leaf N content in geranium, with healthy geraniums having an NDVI greater than 0.8 (Wang et al., 2012). Quijia-Pillajo et al. (2025) reported the effect of phosphate solubilizing bacteria (PSB) on the average GLI, NDVI, NPCI, PSRI of marigold, radish, and tomato grown under P deficiency. Marigolds treated with PSB had higher GLI and lower NPCI and PSRI than untreated marigolds (Quijia-Pillajo et al., 2025). However, that investigation did not include plants grown at optimal fertility.

Plant cultivation in greenhouses demands generous use of synthetic fertilizers to ensure timely production of high market value crops. Vegetation indices have proven to provide accurate information about plant nutritional status in ornamental crops (Osborne et al., 2004; Wang et al., 2012; Jasim et al., 2020). However, reference values representing healthy vs unhealthy ornamental plants are still unavailable in the literature. Leaf tissue nutrient standards have been researched and serve as tools for growers and crop advisors to diagnose nutrient disorders (Krug et al., 2010; Whipker et al., 2024). Similarly, the reference values of vegetation indices could become a tool to diagnose nutrient disorders for researchers and growers. We hypothesized that the spectral signature corresponding to N, P, and K stress is species dependent. Therefore, our study was conducted to determine the value of vegetation indices (GLI, NDVI, NPCI, PSRI) that correspond with N, P, and K deficiency in five horticultural crops.

## 2 Materials and methods

### 2.1 Plant material and growing conditions

The plant species and cultivars included: *Solenostemon scutellarioides* ‘Wizard Gold’ (coleus), *Solanum lycopersicum* ‘Early Girl Plus’ (tomato), *Tagetes patula* ‘Durango Yellow’ (marigold), *Petunia* × *hybrida* ‘Dreams Red’ (petunia), and *Celosia argentea* ‘Glorious Red’ (celosia). Seeds were sown in PRO-MIX PGX substrate (Premier Tech Horticulture, Quakertown, PA, USA), hydrated with RO water, and germinated under propagation domes to provide high humidity. A 12-h photoperiod was provided using LED lights (Arize Lynk LED, Hort Americas, Bedford, TX, USA), and the average temperature range was 20-25 °C. Once cotyledons were fully expanded, seedlings were fertilized with half-strength Hoagland’s solution (Barnes et al., 2012) to provide nutrients needed for seedling development (105 mg L^-1^ N, 15.5 mg L^-1^ P, and 117.3 mg L^-1^ K). Seedlings were transferred to the greenhouse when they had two sets of fully expanded true leaves. After one week in the greenhouse, plants were transplanted into 11.4-cm standard round pots filled with peat-based substrate (80% peat: 20% perlite) (Premier Tech Horticulture, Quakertown, PA, USA; PVP Industries Inc., North Bloomfield, OH, USA) amended with wetting agent (Aqua-Gro L, Aquatrols, Paulsboro, NJ, USA) and dolomitic limestone (calcium carbonate equivalent = 95%; Oldcastle Lawn & Garden, Atlanta, GA, USA) to adjust the pH to 6.2. Prior to transplant, seedlings were leached with RO water to remove nutrients that were not taken up by the plants. Nutrient deficiency treatments began after transplant. The average day temperature was 22.8 ± 1.6 °C with an average relative humidity of 30.2%, and the average night temperature was 16.2 ± 1.5 °C. Supplemental lighting was supplied during the 14-h photoperiod (0600 to 2000 HR) using a 50:50 combination of high-pressure sodium and metal halide lamps when the exterior light intensity fell below 200 µmol m^-2^ s^-1^, while shade curtains were deployed when the exterior light intensity exceeded 300 µmol m^-2^ s^-1^. The average daytime PAR was 162.5 m^-2^ s^-1^.

### 2.2 Nutrient solution treatments

Nutrient deficiency treatments began after transplant. Detailed information on the components and preparation of the nutrient solutions can be found in Barnes et al., 2012. A modified Hoagland’s solution with complete macro and micronutrients was used as the control (Barnes et al., 2012). Nutrient solutions that excluded either nitrogen (-N), phosphorus (-P), or potassium (-K) were used to create specific nutrient-deficient treatments according to Barnes et al., 2012. These nutrient solutions were applied as 100 mL drenches to the peat substrates twice weekly through the duration of the experiments. Plants were irrigated with RO water between nutrient solution treatments as needed. Each nutrient treatment [complete (control), -N, -P, and -K] had eight replicates per plant species (n = 8). The experiment duration varied for each plant species and was based on the development of deficiency symptoms and plant maturity. The experiment duration for coleus, petunia, and celosia was 5 weeks and tomato and marigold was 6 weeks.

### 2.3 Digital phenotyping

The TraitFinder digital phenotyping system (Phenospex, Heerlen, Netherlands) was used to quantify differences in plant growth and health, including nutrient deficiency symptoms. The TraitFinder uses two PlantEye F600 multispectral scanners to create a 3D model of the scanned plants. The 3D model can be represented as a cloud of points, each point representing a specific location on the canopy surface captured by the PlantEye F600 scanners. Each point contains its x, y, and z coordinates along with associated spectral data (red, green, blue, and near-infrared light reflectance). The data was processed by the Phenospex HortControl software to obtain the values of morphological parameters (digital biomass and leaf area), canopy color (hue), and vegetation indices [green leaf index (GLI), normalized difference vegetation index (NDVI), normalized pigment chlorophyll index (NPCI), and plant senescence reflectance index (PSRI)]. Scans were taken weekly for each plant species.

Hue represents the color of each point in the 3D cloud. The hue values unit is degree (°) and represents the color’s position on the color wheel. Hue was used to create a canopy color profile. Using HortControl, we calculated the proportion of the canopy falling into four different hue ranges representing red (300° to 30°), yellow (30° to 90°), green (90° to 180°), and blue (180° to 300°) colors.

### 2.4 Data filtering with the Phenospex HortControl software for digital removal of flower data

Towards the end of the experiment, marigold, petunia, and celosia plants had flowered. The colors of the flowers and the vegetative foliage are included in the reflectance data used to calculate the spectral indices (GLI, NDVI, NPCI, and PSRI). If a dataset representing only the foliage tissue is desired for analysis, it is necessary to either manually remove the flowers from the plant before scanning or to remove data points associated with flower tissue from the 3D model created from plants with flowers (Quijia Pillajo et al., 2023).

HortControl software was used to filter data points associated with flowers from the 3D point cloud based on their spectral information. The distribution of values of all spectral parameters (GLI, NDVI, NPCI, PSRI, Hue, and Saturation) was visually analyzed to identify a range of values tightly associated with flowers that could be used to exclusively filter out flowers and digitally remove them from the 3D point cloud. All spectral variables were evaluated and the threshold range that consistently excluded only floral tissue, while preserving leaf tissue, across samples was selected and applied to all scans before downstream analysis (**Supplementary File 1**). Each of these plant species required a different filtering method to remove the flower data points from the scans. The yellow marigold flowers were filtered by excluding all datapoints with saturation values over 70. Red petunia flowers were filtered by excluding GLI data less than zero. The orange celosia flowers were excluded from scanned data files by filtering out PSRI data over 0.3. To compare spectral results from digital flower exclusion versus manual removal at the end of each experiment, marigold, petunia, and celosia were scanned before and after all flowers and buds with color were manually removed. All parameters were compared between data obtained from digital exclusion of the flowers and manual removal of the flowers. Although these thresholds can serve as an initial reference for cultivars of the same species, they must be redefined for every new cultivar tested, particularly those with distinct flower or canopy colors.

### 2.5 Leaf tissue nutrient analysis

After the final week of nutrient solution treatment and the final scan, leaves from each plant were removed and placed in individual paper bags. The tissue was dried in a forced air-drying oven (60 °C) until completely dried. For tissue nutrient analysis, two replicate plants were combined for a total of four replicates per treatment for each plant species (n = 4). The dried leaf tissue was ground with a ceramic mortar and pestle into a fine powder to pass through a 2-mm sieve. Total nitrogen (N) was measured at Spectrum Analytic (Washington Court House, OH, USA) by combustion analysis using a CN828 Carbon and Nitrogen Analyzer (LECO, St. Joseph, MI, USA). All other mineral nutrients were measured at the Service Testing and Research Laboratory (STAR Lab, The Ohio State University, Wooster, OH, USA), where the dried leaf tissue was digested using the MARS 6 microwave digestion system (CEM Corporation, Matthews, NC, USA). The total concentrations of phosphorus (P), potassium (K), aluminum (Al), boron (B), calcium (Ca), copper (Cu), iron (Fe), magnesium (Mg), manganese (Mn), molybdenum (Mo), sodium (Na), sulfur (S), and zinc (Zn) were obtained from the Agilent 5110 ICP-OES system (Agilent Technologies, Santa Clara, CA, USA) (Isaac and Johnson, 1985).

### 2.6 Statistical analysis

Each plant species was set up as a separate experiment in a randomized complete block design (RCBD) with eight replicates per nutrient solution (n = 8). Means for all measured digital traits along with the *post-hoc* test results are provided as **Supplementary Material** (**Supplementary Tables S1**–**S5**). All statistical analysis and graphs were done in R statistical software (R version 4.3.1) (R Core Team, 2022). Data was analyzed using analysis of variance (ANOVA). When the ANOVA identified significant differences, the mean comparisons were conducted with the Tukey’s HSD test at α = 0.05 (p < 0.05). The Shapiro-Wilk’s test and the Lilliefors test were used to test the normality of the residuals. The Levene’s test and Bartlett test were used to test for the homogeneity of variances.

## 3 Results

### 3.1 Nutrient concentration of leaf tissue from plants grown under nitrogen, phosphorus, and potassium deficiency

Primary (N, P, K) and secondary (Ca, Mg, S) macronutrient concentrations were obtained from leaf tissue of celosia, coleus, marigold, petunia, and tomato plants (**Table 1**). For each species, plants grown with nitrogen (N), phosphorus (P), or potassium (K) deficient Hoagland’s solution displayed reduced concentration of the corresponding nutrient (**Table 1**) in leaf tissue compared to those plants irrigated with complete nutrient solution (control). The N, P, and K levels in plants grown under each respective deficiency were all below the recommended ranges (Gibson et al., 2007; Bryson et al., 2014; Owen et al., 2017; Whipker et al., 2024).

**Table 1:** Primary and secondary macronutrient concentrations in leaves of plant species irrigated with a modified Hoagland’s solution with complete mineral components (this served as the control) or deficient in nitrogen (-N), phosphorus (-P), or potassium (-K).

Hoagland's solution	N (%)	P (%)	K (%)	Ca (%)	Mg (%)	S (%)
Celosia						
Complete	3.7 ± 0.06 B	0.33 ± 0.019 C	3.89 ± 0.15 A	2.17 ± 0.039 A	2.64 ± 0.051 B	0.45 ± 0.023 A
-N	2.2 ± 0.08 C	1.30 ± 0.14 A	4.59 ± 0.28 A	1.41 ± 0.082 C	1.47 ± 0.095 C	0.25 ± 0.0086 C
-P	4.0 ± 0.1 B	0.07 ± 0.0013 D	3.03 ± 0.14 B	1.93 ± 0.11 AB	1.58 ± 0.065 C	0.27 ± 0.0028 C
-K	4.7 ± 0.07 A	0.70 ± 0.011 B	0.68 ± 0.042 C	1.85 ± 0.020 B	3.70 ± 0.044 A	0.39 ± 0.0091 B
Coleus						
Complete	4.1 ± 0.06 A	0.60 ± 0.042 C	4.59 ± 0.11 B	1.81 ± 0.053 B	1.70 ± 0.031 B	0.27 ± 0.0028 AB
-N	2.5 ± 0.4 B	0.91 ± 0.052 B	6.17 ± 0.30 A	1.98 ± 0.065 B	1.64 ± 0.043 B	0.23 ± 0.012 B
-P	3.9 ± 0.1 AB	0.07 ± 0.0034D	3.14 ± 0.16 C	1.74 ± 0.058 B	1.32 ± 0.038 C	0.17 ± 0.0095 C
-K	4.2 ± 0.01 A	1.38 ± 0.052 A	0.72 ± 0.011 D	2.35 ± 0.059 A	2.98 ± 0.020 A	0.29 ± 0.0045 A
Marigold						
Complete	2.9 ± 0.2 B	0.24 ± 0.012 B	2.29 ± 0.12 A	2.07 ± 0.065 B	1.58 ± 0.062 B	0.59 ± 0.020 A
-N	1.0 ± 0.03 C	0.21 ± 0.0041 B	1.06 ± 0.069 C	1.70 ± 0.032 C	0.89 ± 0.022 D	0.40 ± 0.017 B
-P	5.4 ± 0.1 A	0.05 ± 0.0019 C	1.95 ± 0.042 B	2.14 ± 0.061 B	1.19 ± 0.034 C	0.68 ± 0.028 A
-K	3.4 ± 0.1 B	0.69 ± 0.030 A	0.46 ± 0.022 D	2.38 ± 0.039 A	2.70 ± 0.051 A	0.71 ± 0.046 A
Petunia						
Complete	4.0 ± 0.2 B	0.29 ± 0.022 B	4.30 ± 0.31 A	1.87 ± 0.060 A	1.61 ± 0.053 B	0.54 ± 0.018 A
-N	1.7 ± 0.06 C	0.26 ± 0.0060 B	4.64 ± 0.12 A	1.87 ± 0.034 A	1.05 ± 0.030 C	0.41 ± 0.0089 B
-P	5.7 ± 0.2 A	0.05 ± 0.0024 C	3.34 ± 0.11 B	1.72 ± 0.10 AB	1.10 ± 0.030 C	0.39 ± 0.0083 B
-K	4.4 ± 0.04 B	0.84 ± 0.029 A	0.28 ± 0.0072 C	1.52 ± 0.039 B	2.08 ± 0.057 A	0.53 ± 0.017 A
Tomato						
Complete	1.7 ± 0.07 B	0.14 ± 0.0084 C	1.50 ± 0.015 B	1.27 ± 0.059 B	1.07 ± 0.020 C	0.30 ± 0.012 B
-N	1.0 ± 0.02 C	0.23 ± 0.012 B	1.78 ± 0.15 B	1.45 ± 0.078 B	0.82 ± 0.048 D	0.33 ± 0.0092 B
-P	3.6 ± 0.2 A	0.07 ± 0.0021 C	3.66 ± 0.067 A	3.37 ± 0.053 A	2.78 ± 0.0048 A	0.43 ± 0.011 A
-K	1.9 ± 0.06 B	0.43 ± 0.026 A	0.38 ± 0.017 C	1.50 ± 0.061 B	1.54 ± 0.044 B	0.28 ± 0.011 B

Values are the mean nutrient concentration ± standard error (%, n = 4). A different letter represents a significantly different nutrient concentration for that plant species between the nutrient solutions (p < 0.05).

Specifically, -N treatment significantly reduced N concentration in the leaves of celosia by 41%, coleus by 39%, marigold by 66%, petunia by 58%, and tomato by 41% (**Table 1**). Moreover, -P treatment significantly reduced leaf P concentration by 79% in celosia, 88% in coleus, 79% in marigold, and 83% in petunia compared to control plants (**Table 1**). In tomato, P concentration was reduced by 50% under -P conditions, though this difference was not statistically significant. Finally, -K treatment resulted in significant reductions in leaf K concentration across all species. Compared to control plants, K concentrations were reduced by 84% in celosia, 84% in coleus, 80% in marigold, 93% in petunia, and 75% in tomato (**Table 1**). Micronutrient concentrations were also measured in the leaf tissue, but no consistent patterns emerged among the nutrient deficiency treatments (**Table 2**).

**Table 2:** Micronutrient concentrations in leaves of plant species irrigated with a modified Hoagland’s solution with complete mineral components (this served as a control) or deficient in nitrogen (-N), phosphorus (-P), or potassium (-K).

Hoagland's solution	B (µg·g^-1^)	Cu (µg·g^-1^)	Fe (µg·g^-1^)	Mn (µg·g^-1^)	Mo (µg·g^-1^)	Zn (µg·g^-1^)
Celosia						
Complete	43.5 ± 1.7 B	3.4 ± 0.4 C	103.9 ± 3.6 C	318.6 ± 6.5 B	1.2 ± 0.3 A	97.7 ± 2.7 B
-N	44.0 ± 2.9 AB	7.1 ± 0.8 B	137.0 ± 8.4 AB	602.5 ± 46.0 A	0.9 ± 0.3 A	153.2 ± 11.3 A
-P	44.9 ± 2.7 AB	11.3 ± 0.3 A	162.2 ± 7.2 A	304.7 ± 14.3 B	1.0 ± 0.3 A	110.8 ± 3.3 B
-K	55.1 ± 1.0 A	2.6 ± 0.1 C	113.7 ± 1.2 BC	318.6 ± 7.2 B	0.5 ± 0.03 A	85.2 ± 2.9 B
Coleus						
Complete	33.4 ± 1.0 A	1.9 ± 0.1 B	112.9 ± 3.5 A	122.3 ± 7.4 B	0.7 ± 0.2 A	27.9 ± 0.9 BC
-N	35.1 ± 1.4 A	2.4 ± 0.1 B	137.0 ± 5.9 A	177.5 ± 13.5 A	0.6 ± 0.08 A	29.6 ± 0.8 AB
-P	34.6 ± 1.7 A	4.4 ± 0.3 A	122.0 ± 51.4 A	75.5 ± 5.0 C	0.5 ± 0.07 A	24.8 ± 1.2 C
-K	38.0 ± 1.5 A	1.8 ± 0.06 B	87.6 ± 1.4 A	132.4 ± 1.1 B	0.4 ± 0.2 A	32.9 ± 1.0 A
Marigold						
Complete	60.2 ± 2.3 A	1.6 ± 0.06 C	73.1 ± 4.0 B	187.3 ± 9.6 B	0.4 ± 0.2 B	27.7 ± 1.3 B
-N	27.0 ± 1.3 B	0.6 ± 0.05 D	25.9 ± 0.6 C	95.2 ± 1.8 C	0.2 ± 0.04 B	14.5 ± 0.8 C
-P	60.7 ± 3.8 A	4.4 ± 0.2 A	83.9 ± 3.2 AB	98.4 ± 4.3 C	0.6 ± 0.06 AB	37.7 ± 2.9 A
-K	56.4 ± 1.7 A	2.2 ± 0.1 B	91.5 ± 4.2 A	242.0 ± 7.5 A	1.2 ± 0.2 A	31.0 ± 0.9 AB
Petunia						
Complete	34.0 ± 0.7 B	1.1 ± 0.2 B	130.2 ± 3.5 A	145.6 ± 2.6 B	0.9 ± 0.1 A	32.5 ± 0.5 B
-N	20.5 ± 0.6 C	0.8 ± 0.07 B	123.2 ± 9.0 A	205.4 ± 3.1 A	0.4 ± 0.05 A	55.2 ± 1.4 A
-P	39.9 ± 1.5 A	5.5 ± 0.5 A	185.3 ± 33.8 A	123.6 ± 5.9 C	1.1 ± 0.1 A	62.3 ± 2.6 A
-K	44.0 ± 1.7 A	1.5 ± 0.09 B	200.8 ± 44.4 A	124.3 ± 4.7 C	0.3 ± 0.09 A	34.6 ± 2.7 B
Tomato						
Complete	33.8 ± 0.4 A	0.7 ± 0.02 B	47.1 ± 7.1 BC	64.3 ± 2.4 C	0.3 ± 0.2 AB	11.6 ± 0.2 C
-N	34.0 ± 0.3 A	0.5 ± 0.1 B	30.8 ± 2.9 C	118.5 ± 5.5 B	0.3 ± 0.05 B	24.8 ± 1.7 B
-P	34.6 ± 1.0 A	6.9 ± 0.3 A	162.8 ± 5.7 A	461.4 ± 17.6 A	0.9 ± 0.1 A	60.2 ± 1.2 A
-K	31.5 ± 1.3 A	0.6 ± 0.04 B	54.2 ± 2.8 B	52.1 ± 0.9 C	0.4 ± 0.1 AB	14.8 ± 1.8 C

Values are the mean nutrient concentration ± standard error (µg·g^-1^, n = 4). A different letter represents a significantly different nutrient concentration for that plant species compared between the nutrient solutions (p-value < 0.05).

### 3.2 Digital phenotyping of flowering ornamentals

Marigold, petunia, and celosia plants began flowering in week 4, so scans of the shoots at week 4 and beyond included spectral information from both the foliage and the flowers. Spectral data from flower tissue can differ from vegetative foliage tissue, which can skew calculated health parameters (Ollinger, 2011; Van Der Kooi et al., 2016; Quijia Pillajo et al., 2023). HortControl software enables the filtering of data points from the 3D cloud based on spectral information. Thus, spectral data can be utilized to remove data points from the 3D cloud that correspond with flower tissue. Since we were interested in the spectral response of foliage, flowers were removed from the dataset using HortControl. Flower data points were filtered out from the scans using the spectral parameter values (GLI, NDVI, NPCI, PSRI, Hue, or Saturation) that differentiated foliage from flowers. The distribution of values of each spectral parameter was analyzed to find ranges closely associated with flowers. For instance, we observed that data points corresponding to marigold flowers had saturation values from 70 to 100, data points corresponding to petunia flowers had GLI values < 0, and data points corresponding to celosia flowers had PSRI values > 0.3. We visually identified the spectral parameter that most effectively removed flower data points while minimizing its impact on foliage-related data points. The selected filtering parameters to remove flowers were species dependent: saturation > 70 for marigold, GLI < 0 for petunia, and PSRI > 0.3 for celosia.

**Figure 1:**
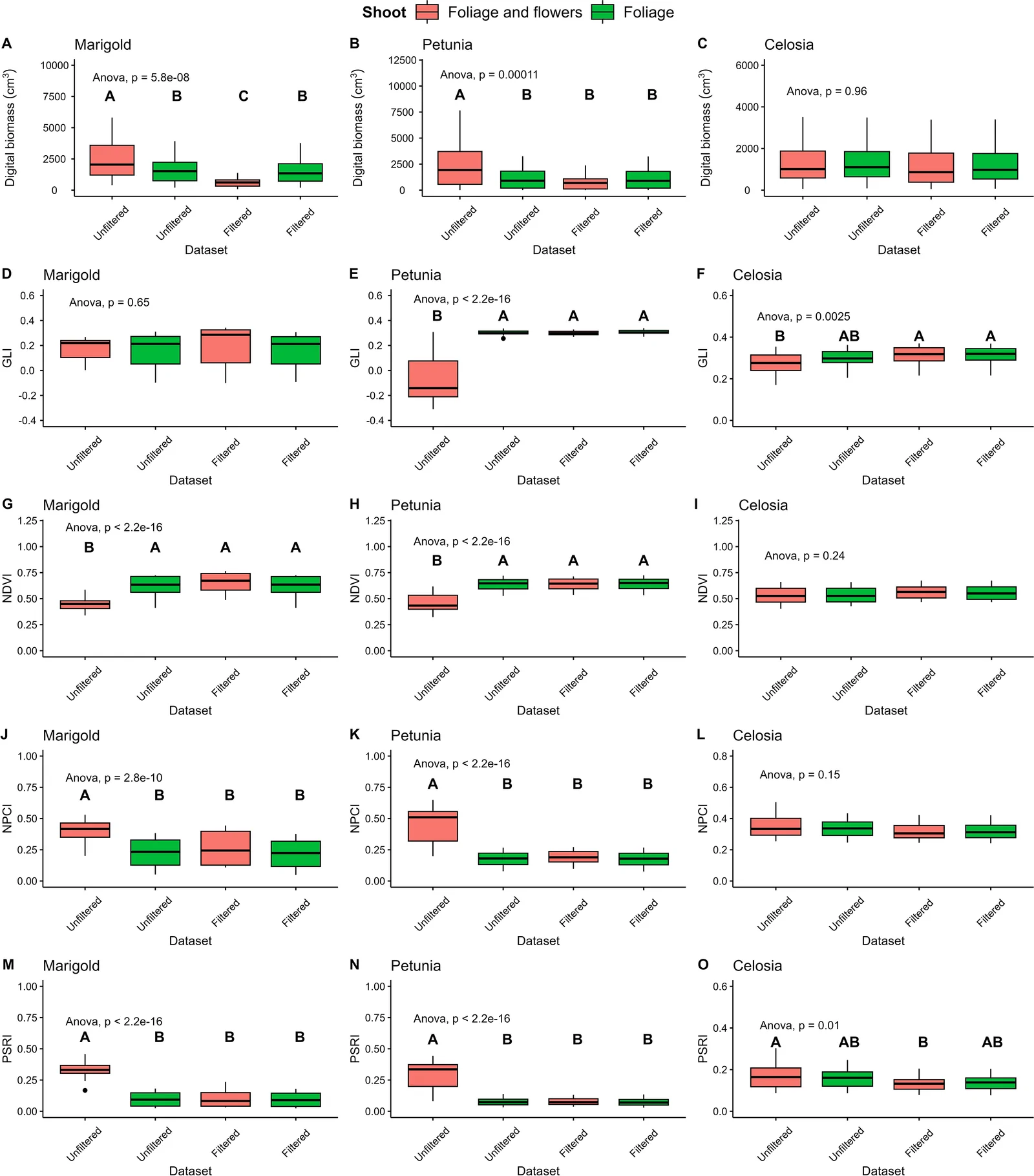
Comparison of morphological and spectral parameters from plants that have had flowers physically removed from the plant or digitally filtered from the 3D images. Digital phenotyping parameters of marigold **(A, D, G, J, M)**, petunia **(B, E, H, K, N)**, and celosia **(C, F, I, L, O)**. Three dimensional (3D) scanned images of plants with flowers intact (pink boxes, foliage and flowers) and after manual removal of flowers (green boxes, foliage). A filter was applied to remove flower tissue from the original foliage and flowers dataset. The same filter was applied to the original foliage dataset to confirm that the filtering was specifically removing only flowers and not foliage. Parameters evaluated include digital biomass **(A–C)**, GLI (green leaf index, **(D–F)**), NDVI (normalized difference vegetation index, **(G–I)**), NPCI (normalized pigment chlorophyll index, **(J–L)**), and PSRI (plant senescence reflectance index, **(M–O)**). For each plot, boxes represent the interquartile range, the line within the box represents the median, and whiskers extend to the minimum and maximum value for each data set. Different letters represent significant differences between the datasets, with the p-value listed in each plot.

To evaluate the effectiveness of our data filtering approach, we compared the scans taken at the end of each experiment from plants before (original foliage-flowers dataset) and after (original foliage) manual flower removal. The dataset collected before flower removal (original foliage-flower dataset) included both foliage and flowers, while the dataset collected after manual flower removal (original foliage dataset) included foliage only. The original foliage dataset served as our reference, because it provided the most accurate representation of foliage digital biomass and spectral indices. As expected, the presence of flowers greatly influenced the values of the digital biomass and vegetation indices (**Figure 1**). We applied the selected flower filtering parameters to the original foliage-flowers and original foliage datasets, which resulted in two additional datasets (filtered foliage-flowers and filtered foliage). Analysis of variance was performed to compare the original and filtered datasets for digital biomass, GLI, NDVI, NPCI, and PSRI (**Figure 1**). We found a significant effect of flowers in the datasets of marigold and petunia (**Figure 1**). Although present, the flower effect on celosia data was less than on marigold and petunia data, which could be associated with the small size of the celosia flowers present primarily in the control treatment.

As expected, in marigold and petunia, digital biomass in the original foliage-flowers dataset was higher than in the original foliage dataset (**Figures 1A, B**). Digital biomass and leaf area can be impacted by filtering when flowers are present, because the TraitFinder does not capture areas of the canopy shaded or occluded by the flowers. After filtering, the canopy area beneath the flowers is excluded because data points for that area were not detectable to the scanner. For instance, in marigold, digital biomass in the filtered foliage-flowers dataset was lower than in the original foliage dataset, but the filtered and original foliage datasets were not different (**Figure 1A**). The flowers occluded the leaf canopy from the scan, resulting in an underrepresentation of the shoot digital biomass. Therefore, for flowering species, growth evaluations (digital biomass and leaf area) were based on the unfiltered dataset. Analysis of growth parameters for all plant species using the filtered data are provided as supplementary files (**Supplementary Tables S1**–**S5**).

Importantly, the results clearly showed a difference in all vegetation indices (GLI, NDVI, NPCI, and PSRI) between the original foliage-flowers dataset and the original foliage dataset (**Figures 1E, G, H, J, K, M, N**). In contrast, the filtered foliage-flowers dataset did not differ from the original foliage dataset, indicating that our filtering approach successfully removed the flowers. We found no differences between the original foliage dataset and the filtered foliage dataset, further suggesting that our filtering approach removed flowers without affecting the foliage (**Figures 1E, G, H, J, K, M, N**). Thus, our filtering approach effectively eliminated the flower presence bias from the vegetation indices data. Given the impact of flowers on vegetation indices, we evaluated foliage health for this manuscript using the filtered datasets. Analysis of vegetation indices with unfiltered data are provided as Supplementary Files (**Supplementary Tables S1**–**S5**).

### 3.3 Digital phenotyping of coleus plants grown under nitrogen, phosphorus, and potassium deficiency

**Figure 2:**
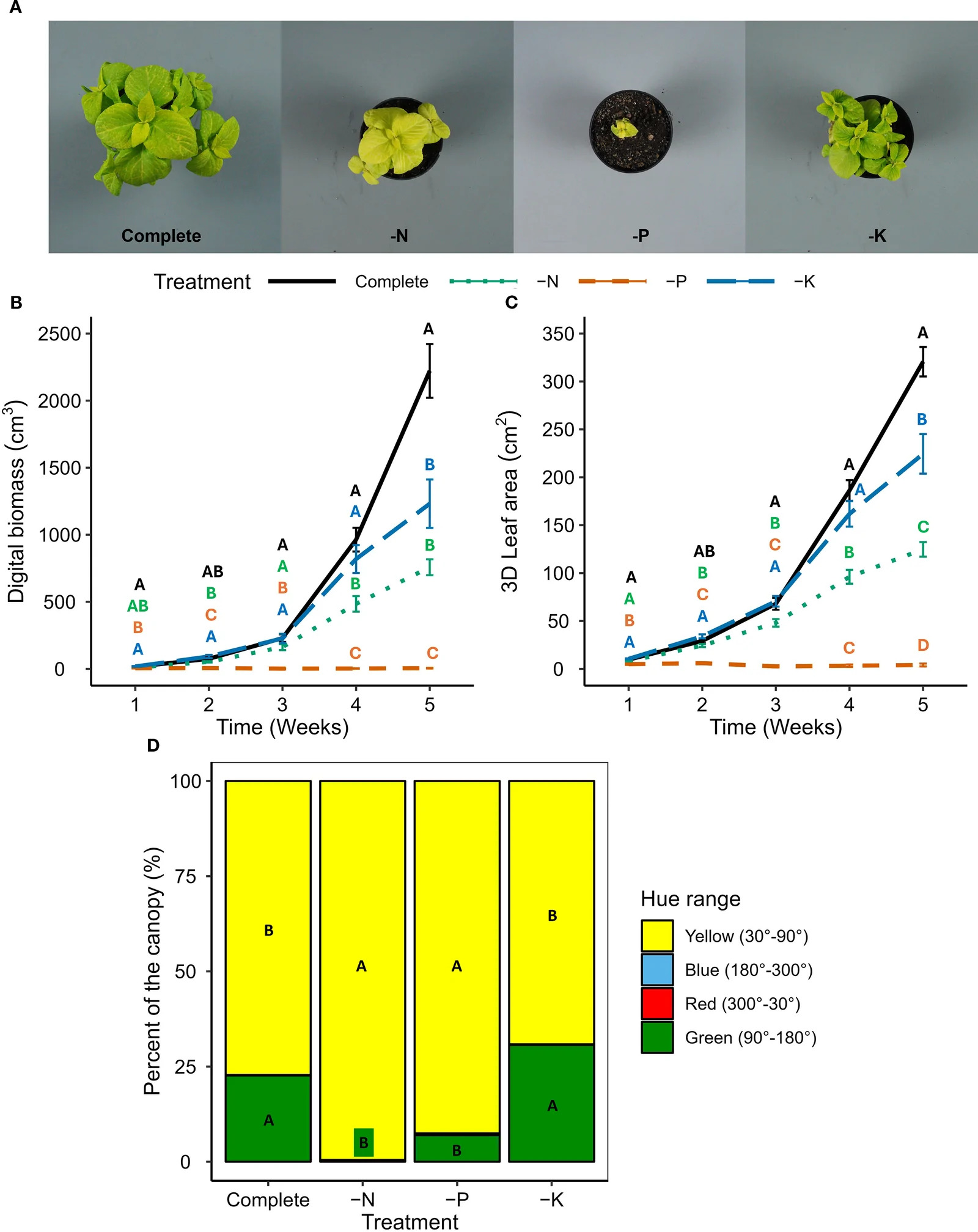
Coleus plants were grown with modified Hoagland’s solution containing all nutrients (complete, control) or modified Hoagland’s solutions deficient in nitrogen (-N), phosphorus (-P), or potassium (-K) and evaluated for morphological differences. Representative coleus plants five weeks from treatment initiation **(A)**. Digital biomass **(B)** and 3D leaf area **(C)** of coleus plants irrigated with the different nutrient solutions were evaluated weekly. Lines represent the nutrient solution treatments and indicate the mean measured value for each week; error bars represent the standard error (n = 8), and different letters indicate significant differences between nutrient solution treatments at each week (p < 0.05). Plant foliage color was evaluated with spectral reflectance data, where the percentage of the leaf canopy associated with each hue range after five weeks is presented **(D)**. Letters represent significant differences between treatments for each hue range (n = 8, p < 0.05). All data is from unfiltered 3D scanned images.

Nitrogen (N), phosphorus (P), and potassium (K) deficient Hoagland’s solutions limited growth of coleus, with the most extreme stunting seen under P deficiency (**Figure 2A**). Differences in growth are represented as digital biomass and 3D leaf area (**Figures 2B, C**). The effect of N and K deficiency on coleus growth appeared later in the experiment, at week 4 and week 5, respectively. In contrast, growth limitation was observed from week 1 in P deficient coleus. At week 5, the average digital biomass of N, P, and K deficient coleus was 66%, 99%, and 45% less than control (complete nutrient solution) coleus, respectively (**Figure 2B**). Similarly, the 3D leaf area of coleus plants deficient in N, P, and K was reduced by 61%, 99%, and 30%, respectively, compared to control plants (**Figure 2C**).

The average color profile of coleus was evaluated on the scan made on week 5. The average color profile of control coleus plants irrigated with the complete Hoagland’s solution was 77.19% yellow, 0.05% blue, 0.10% red, and 22.66% green (**Figure 2D**). In the N and P deficient coleus, the proportion of green foliage decreased to 0.24% and 6.98%, and the proportion of yellow foliage increased to 99.54% and 92.55%, respectively. In K deficient coleus, the proportion of green increased to 30.66% and the proportion of yellow decreased to 69.16% (**Figure 2D**). A reduction in the proportion of red and blue foliage was only observed in the P deficient plants (**Figure 2D**).

**Figure 3:**
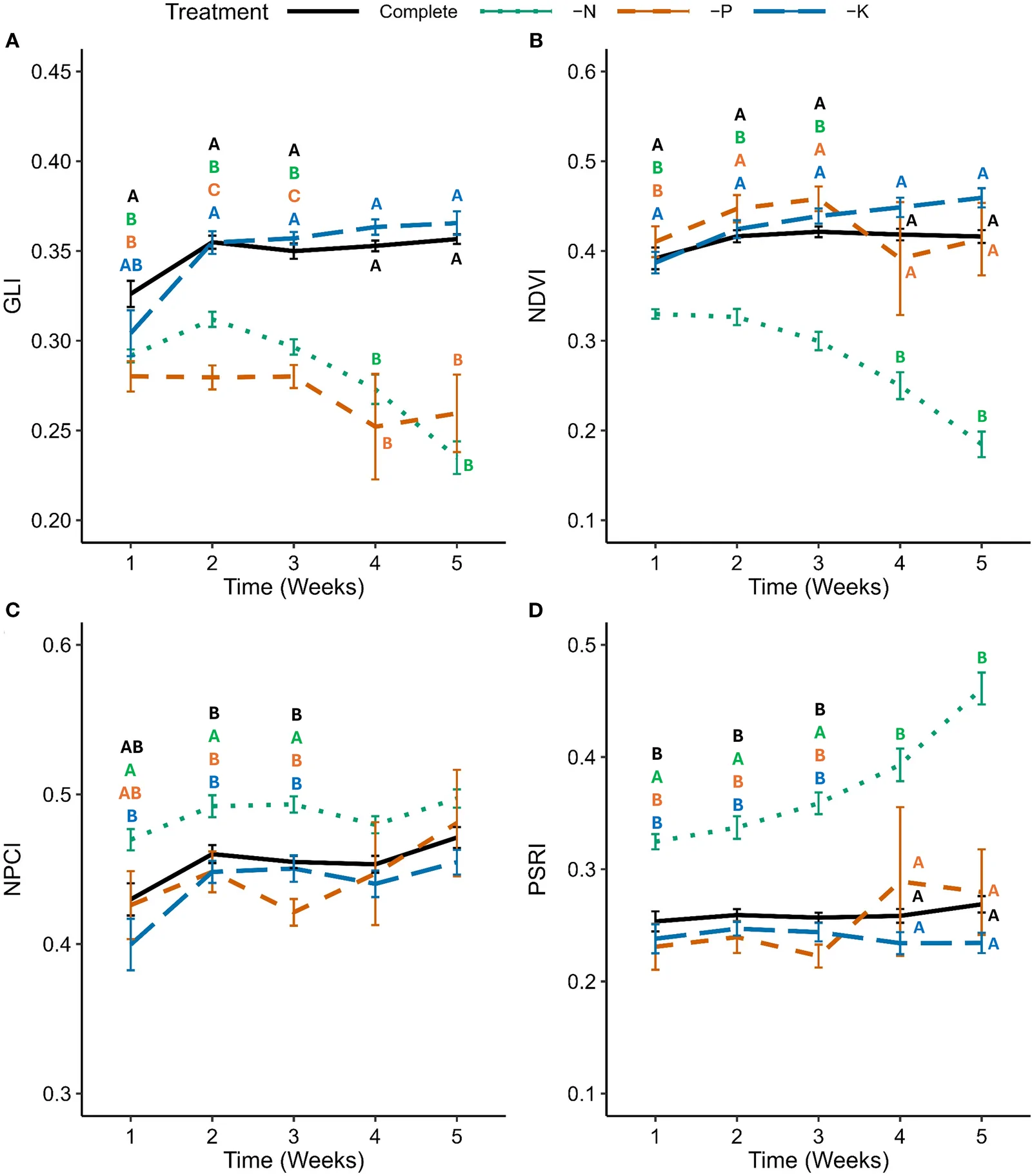
Coleus plants were grown with modified Hoagland’s solution containing all nutrients (complete, control) or modified Hoagland’s solutions deficient in nitrogen (-N), phosphorus (-P), or potassium (-K) and evaluated weekly for differences in vegetation indices using unfiltered 3D scanned images. The indices measured include: GLI [green leaf index, **(A)**], NDVI [normalized difference vegetation index, **(B)**], NPCI [normalized pigment chlorophyll index, **(C)**], and PSRI [plant senescence reflectance index, **(D)**]. For each plot, lines represent the nutrient solution treatments and indicate the mean index for each week; error bars represent the standard error (n = 8), and letters represent significant differences between nutrient solution treatments at each week (p < 0.05).

Values for the vegetation indices of coleus grown with complete Hoagland’s (control) and macronutrient deficiencies (-N, -P, and -K) are presented in **Figure 3**. Average vegetative indices for control coleus plants irrigated with complete Hoagland’s solution ranged from 0.33 to 0.36 for GLI, 0.39 to 0.42 for NDVI, 0.43 to 0.47 for NPCI, and 0.25 to 0.27 for PSRI (**Figure 3**).

All vegetation indices quantitatively distinguished N-deficient coleus plants from control plants (complete Hoagland’s). Compared to control plants, coleus under N deficiency showed lower GLI and NDVI and higher NPCI and PSRI (**Figures 3A, B**). By week 5, the GLI and NDVI values for N-deficient plants were 34% and 56% lower than those of control plants, respectively (**Figures 3A, B**), while the PSRI was 72% higher (**Figure 3D**). NPCI was only affected by N deficiency in weeks 2 and 3 (**Figure 3C**). P-deficient coleus plants were only distinguished from the control by GLI values, which were significantly lower than control plants from week 1 through week 5 (**Figure 3A**). The GLI, NDVI, NPCI, and PSRI of control and K-deficient coleus were not different (**Figure 3**).

### 3.4 Digital phenotyping of tomato plants grown under nitrogen, phosphorus, and potassium deficiency

**Figure 4:**
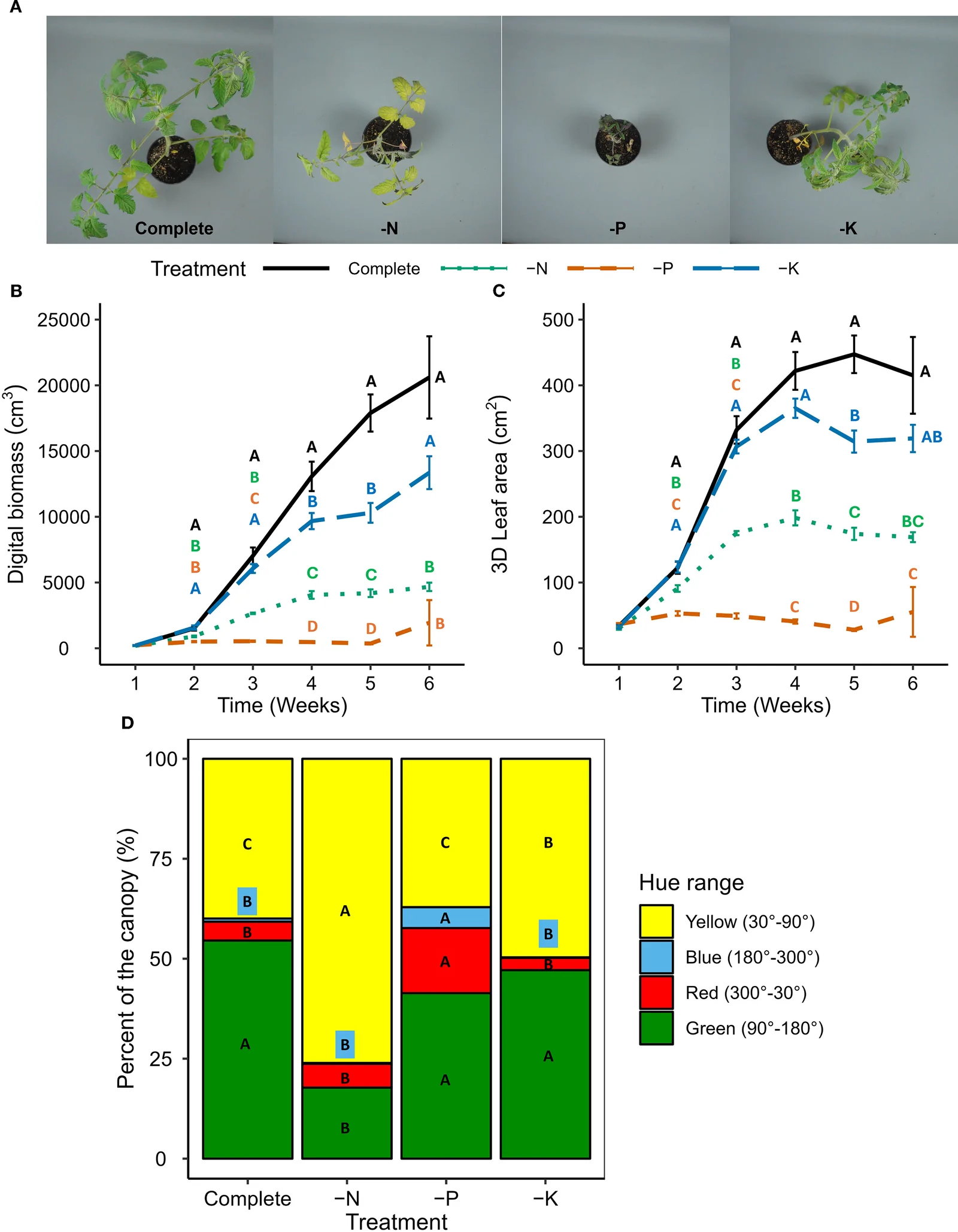
Tomato plants were grown with modified Hoagland’s solution containing all nutrients (complete, control) or modified Hoagland’s solutions deficient in nitrogen (-N), phosphorus (-P), or potassium (-K) and evaluated for morphological differences. Representative tomato plants six weeks from treatment initiation **(A)**. Digital biomass **(B)** and 3D leaf area **(C)** of tomato plants irrigated with the different nutrient solutions were evaluated weekly. Lines represent the nutrient solution treatments and indicate the mean measured value for each week; error bars represent the standard error (n = 8), and different letters indicate significant differences between nutrient solution treatments at each week (p < 0.05). Plant foliage color was evaluated with spectral reflectance data, where the percentage of the leaf canopy associated with each hue range after six weeks is presented **(D)**. Letters represent significant differences between treatments for each hue range (n = 8, p < 0.05). All data is from unfiltered 3D scanned images.

Nitrogen (N), phosphorus (P), and potassium (K) deficient Hoagland’s solution limited growth of tomato plants, with the most extreme effects observed under P deficiency (**Figure 4A**). Differences in growth are represented as digital biomass and 3D leaf area (**Figures 4B, C**). The effect of N and P deficiency on tomato growth appeared early in the experiment (week 2). In contrast, growth limitation was observed from week 4 in K deficient tomatoes. At week 4, average digital biomass of N, P, and K deficient tomatoes was reduced by 69%, 96%, and 26%, respectively, compared to the control tomatoes that were provided with complete nutrient solution (**Figure 4B**). At week 5, the digital biomass of N, P, and K deficient tomatoes was reduced by 77%, 98%, and 42%, respectively, compared to the control plants (**Figure 4B**). A similar trend was observed with 3D leaf area on week 5 (**Figure 4C**). At week 6, only the digital biomass of N and P deficient tomatoes was significantly different (77% and 91% lower, respectively) than the control tomatoes (**Figure 4B**). Similarly, the 3D leaf area of N and P deficient tomatoes at week 6 was 59% and 87% less than control plants, respectively (**Figure 4C**).

The average color profile (hue) of tomatoes was evaluated on the scan made on week 6. The average color profile of control tomato plants irrigated with the complete Hoagland’s solution was 39.95% yellow, 0.79% blue, 4.71% red, and 54.55% green (**Figure 4D**). In the N deficient tomatoes, the proportion of green foliage decreased to 17.75%, and the proportion of yellow foliage increased to 76.09%. Only P deficient solution increased the proportion of red (16.28%) and blue (5.28%) foliage. In K deficient tomatoes, the only significant difference from the control treatment was an increase in the proportion of yellow to 49.65% (**Figure 4D**).

**Figure 5:**
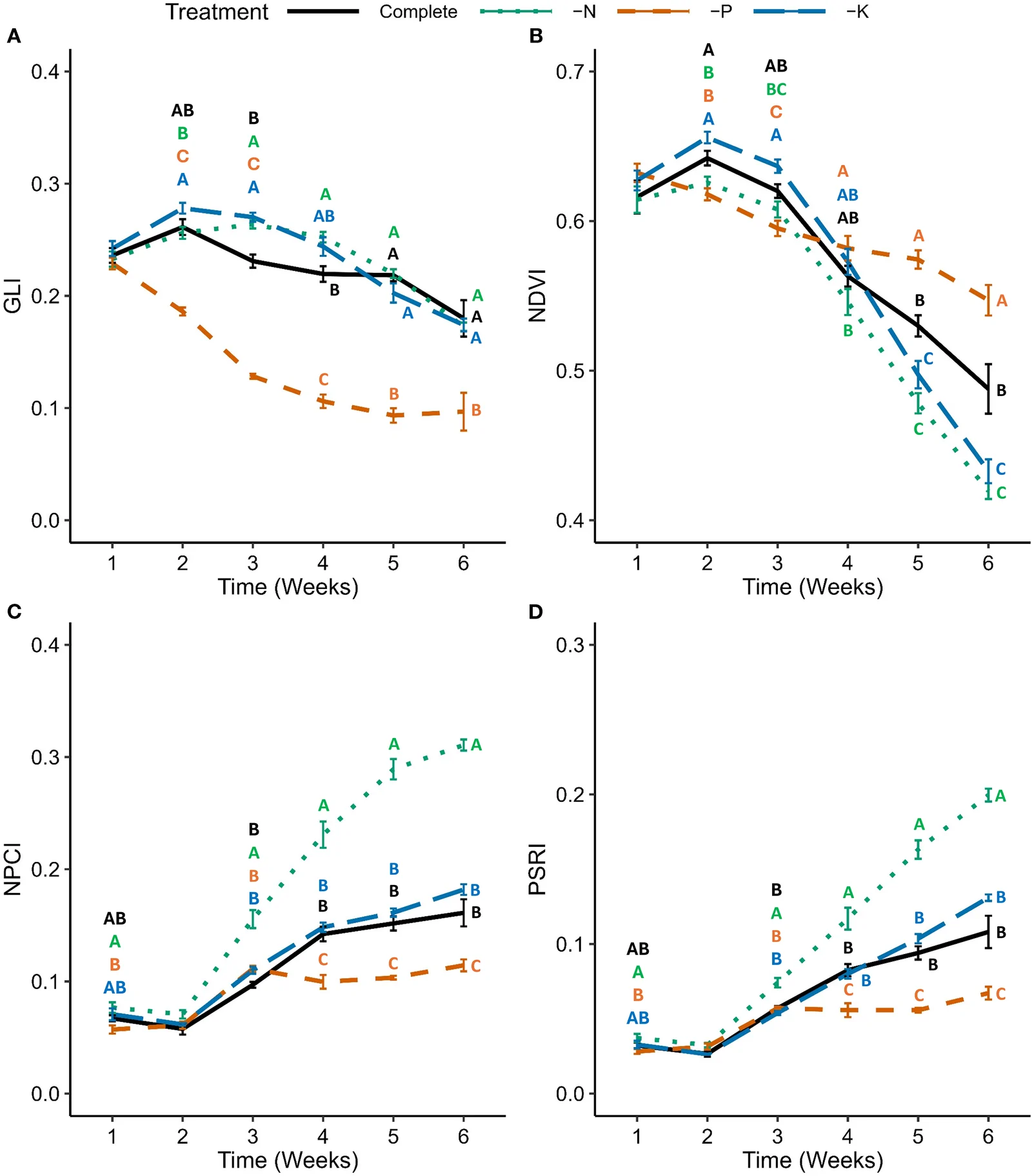
Tomato plants were grown with modified Hoagland’s solution containing all nutrients (complete, control) or modified Hoagland’s solutions deficient in nitrogen (-N), phosphorus (-P), or potassium (-K) and evaluated weekly for differences in vegetation indices using unfiltered 3D scanned images. The indices measured include: GLI [green leaf index **(A)**], NDVI [normalized difference vegetation index **(B)**], NPCI [normalized pigment chlorophyll index **(C)**], and PSRI [plant senescence reflectance index, **(D)**]. For each plot, lines represent the nutrient solution treatments and indicate the mean index for each week; error bars represent the standard error (n = 8), and letters represent significant differences between nutrient solution treatments at each week (p < 0.05).

Vegetation indices of tomatoes irrigated with complete nutrients (control), N, P, or K deficient solutions are presented in **Figure 5**. Tomatoes irrigated with the complete Hoagland’s solution had average GLI, NDVI, NPCI, and PSRI values ranging from 0.18 to 0.26, 0.49 to 0.64, 0.06 to 0.16, and 0.03 to 0.11, respectively (**Figure 5**).

N deficient tomatoes had a similar GLI to control plants that received complete Hoagland’s solution, with slightly higher values only in week 3 and 4 (**Figure 5A**). NDVI of N deficient tomatoes was similar to control plants from week 1 to 4, but it was significantly lower at weeks 5 and 6 (**Figure 5B**). N deficient tomatoes had higher NPCI and PSRI than the control tomatoes starting on week 3 (**Figures 5C, D**). By week 6, the NDVI of N deficient tomatoes was 14% lower than the control plants, and NPCI and PSRI values were 93% and 85% higher than the control plants (**Figures 5C, D**). P deficient tomatoes showed higher NDVI (**Figure 5B**) and lower GLI, NPCI, and PSRI than the control tomatoes (**Figures 5A, C, D**). P deficient tomato plants showed lower GLI from week 2 to 6, and lower NPCI and PSRI than control plants from week 4 to 6 (**Figures 5A, C, D**). In contrast, NDVI of P deficient tomatoes was greater than the control tomatoes at week 5 and 6. (**Figure 5B**). By week 6, the GLI, NPCI, and PSRI values of P deficient tomatoes were 60%, 29%, and 38% lower than the control, respectively. In contrast, the NDVI of P deficient tomatoes was 12% higher than the control (**Figure 5B**). Finally, K deficiency only had a significant effect on NDVI. The NDVI of K deficient tomatoes was 6% and 11% lower than the control on week 5 and 6, respectively (**Figure 5B**).

### 3.5 Digital phenotyping of marigold plants grown under nitrogen, phosphorus, and potassium deficiency

**Figure 6:**
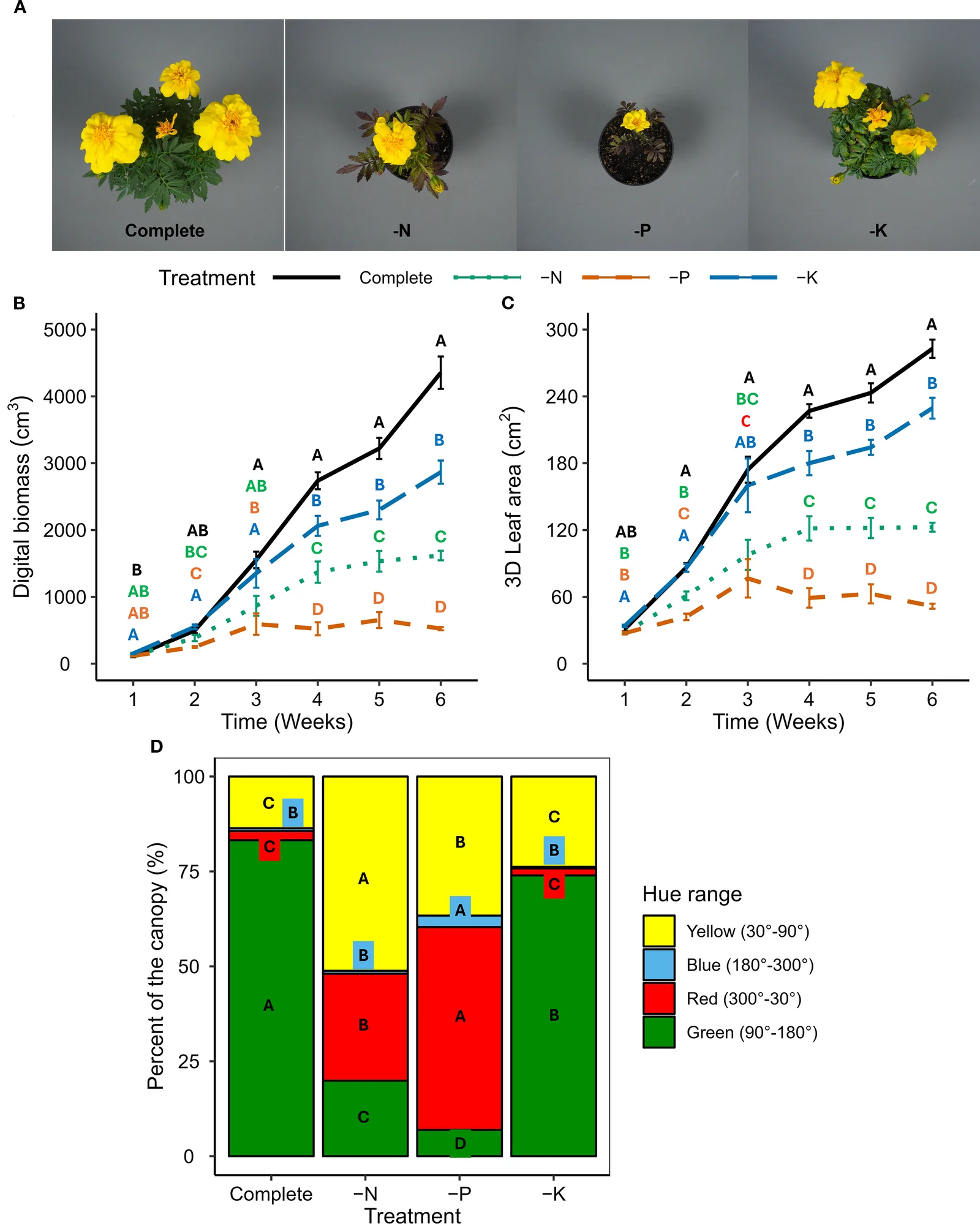
Marigold plants were grown with modified Hoagland’s solution containing all nutrients (complete, control) or modified Hoagland’s solutions deficient in nitrogen (-N), phosphorus (-P), or potassium (-K) and evaluated for morphological differences. Representative marigolds six weeks from treatment initiation **(A)**. Digital biomass **(B)** and 3D leaf area **(C)** of marigolds irrigated with the different nutrient solutions were evaluated weekly. Data is from unfiltered 3D scanned images. Lines represent the nutrient solution treatments and indicate the mean measured value for each week; error bars represent the standard error (n = 8), and different letters indicate significant differences between nutrient solution treatments at each week (p < 0.05). Plant foliage color was evaluated with spectral reflectance data, where the percentage of the leaf canopy associated with each hue range after six weeks is presented **(D)**. Data is from unfiltered 3D scanned images taken after manually removing all flowers. Letters represent significant differences between treatments for each hue range (n = 8, p < 0.05).

Plant growth (digital biomass and leaf area) was evaluated only using the unfiltered dataset (original foliage-flower dataset). Nitrogen (N), phosphorus (P), and potassium (K) deficient Hoagland’s solution limited marigold growth compared to the control marigold plants that were provided complete Hoagland’s solution (**Figure 6A**). Differences in growth are represented as digital biomass and 3D leaf area of plants with flowers (**Figures 6B, C**). The effect of N and K deficiency on marigold growth was detected at week 4, while reductions in growth of P deficient marigold started early in the experiment (week 2 through week 6). At week 5, the average digital biomass of N, P, and K deficient marigolds was 64%, 89%, and 38% less than the control marigolds, respectively (**Figure 6B**). Similarly, the 3D leaf area of N, P, and K deficient marigolds at week 5 was 63%, 84%, and 24% less than control marigolds, respectively (**Figure 6C**).

The average color profile (hue) of marigolds was evaluated on the scan made on week 6 after manually removing all flowers. The color profile of control marigold plants irrigated with the complete Hoagland’s solution was 13.65% yellow, 0.71% blue, 2.42% red, and 83.22% green (**Figure 6D**). In the N deficient marigolds, the proportion of green foliage decreased to 19.87%, and the proportion of yellow and red foliage increased to 51.17% and 28.18%, respectively. In the P deficient marigolds, the proportion of green foliage decreased to 6.92%, and the proportion of yellow, blue, and red foliage increased to 36.64%, 3.02%, and 53.43%, respectively. In K deficient marigolds, only the proportion of green significantly decreased to 73.94% (**Figure 6D**).

**Figure 7:**
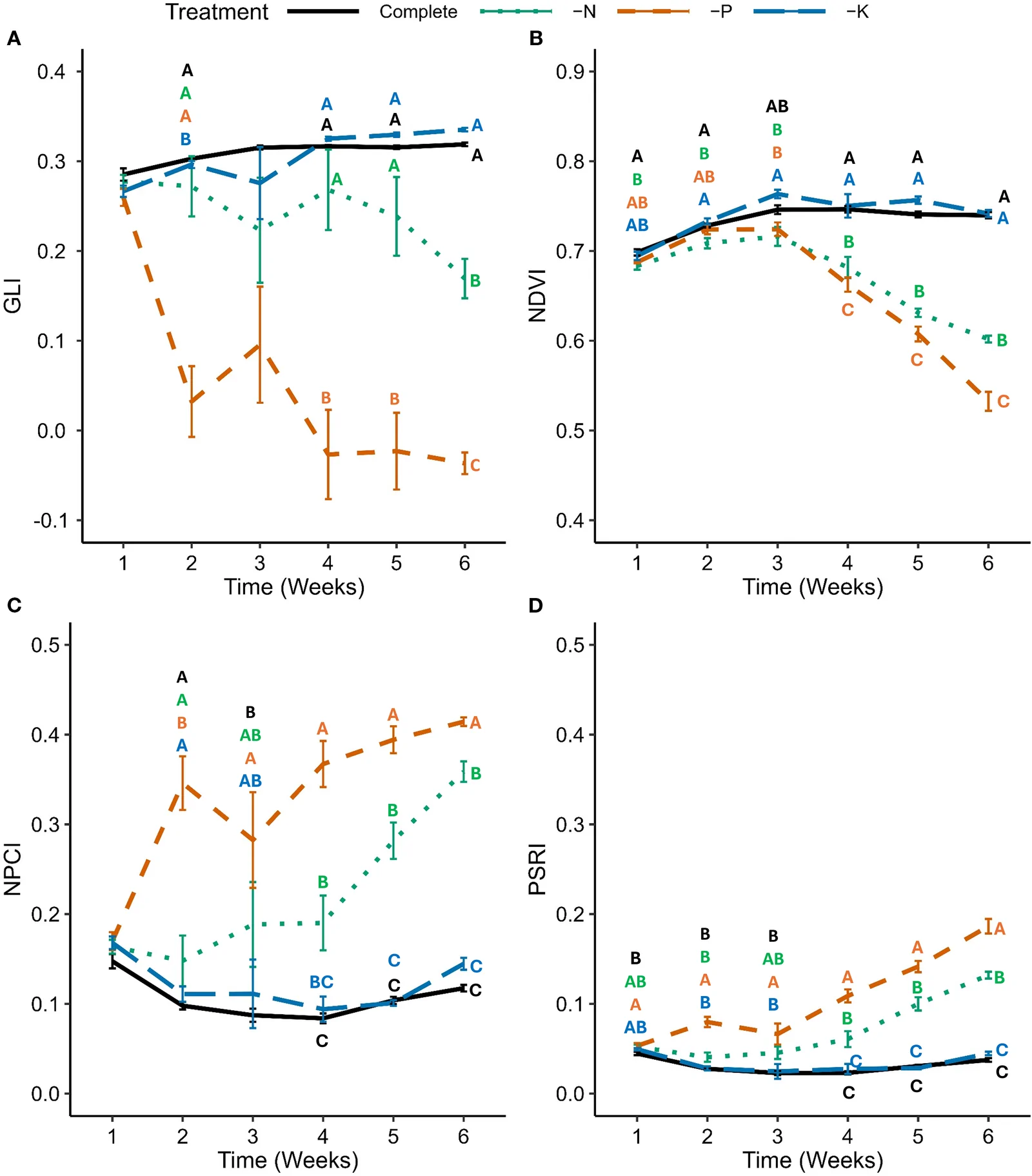
Marigolds were grown with modified Hoagland’s solution containing all nutrients (complete, control) or modified Hoagland’s solutions deficient in nitrogen (-N), phosphorus (-P), or potassium (-K) and evaluated weekly for differences in vegetation indices. Saturation filtering of the 3D scanned images was used to digitally exclude all flower tissue from the dataset. The indices measured include: GLI [green leaf index, **(A)**], NDVI [normalized difference vegetation index, **(B)**], NPCI [normalized pigment chlorophyll index, **(C)**], and PSRI [plant senescence reflectance index, **(D)**]. For each plot, lines represent the nutrient solution treatments and indicate the mean index for each week, error bars represent the standard error (n = 8), and letters represent significant differences between nutrient solution treatments at each week (p < 0.05).

Because of the influence of flower tissue on vegetation indices, we evaluated marigold foliage health using the saturation filtered dataset. All vegetation indices proved to be good indicators of N and P deficiencies (**Figure 7**). Average vegetative indices for marigolds irrigated with complete Hoagland’s solution ranged from 0.25 to 0.32 for GLI, from 0.70 to 0.75 for NDVI, from 0.07 to 0.15 for NPCI, and from 0.02 to 0.04 for PSRI (**Figure 7**). While GLI, NDVI, NPCI, and PSRI remained consistent in the control plants throughout the experiment, GLI and NDVI decreased (**Figures 7A, B**), and NPCI and PSRI increased in N and P deficient marigolds (**Figures 7C, D**). At 6 weeks, the GLI and NDVI values of N deficient marigolds were 53% and 21% lower than the control plants, and the NPCI and PSRI were 413% and 407% higher than the control plants, respectively. At 6 weeks, the GLI and NDVI values of P deficient marigolds were 108% and 30% lower than the control marigolds, and the NPCI and PSRI were 374% and 457% higher than the control, respectively. K deficiency did not affect GLI, NDVI, NPCI, or PSRI for marigolds (**Figures 7C, D**).

### 3.6 Digital phenotyping of petunia plants grown under nitrogen, phosphorus, and potassium deficiency

**Figure 8:**
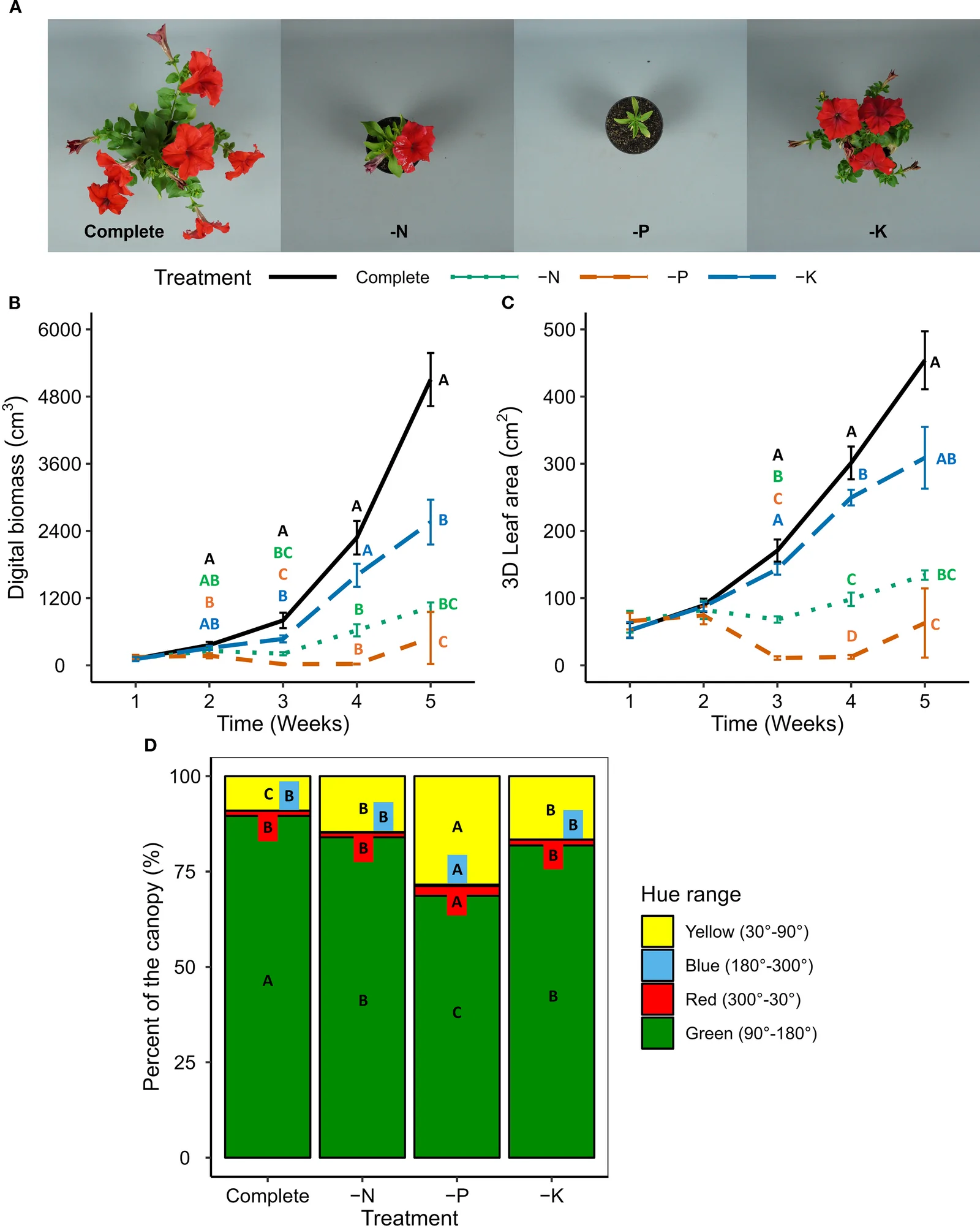
Petunias were grown with modified Hoagland’s solution containing all nutrients (complete, control) or modified Hoagland’s solutions deficient in nitrogen (-N), phosphorus (-P), or potassium (-K) and evaluated for morphological differences. Representative petunia five weeks from treatment initiation **(A)**. Digital biomass **(B)** and 3D leaf area **(C)** of petunia plants irrigated with the different nutrient solutions were evaluated weekly. Data is from unfiltered 3D scanned images. Lines represent the nutrient solution treatments and indicate the mean measured value for each week; error bars represent the standard error (n = 8), and different letters indicate significant differences between nutrient solution treatments at each week (p < 0.05). Plant foliage color was evaluated with spectral reflectance data, where the percentage of the leaf canopy associated with each hue range after five weeks is presented **(D)**. Data is from unfiltered 3D scanned images taken after manually removing all flowers. Letters represent significant differences between treatments for each hue range (n = 8, p < 0.05).

Petunia growth (digital biomass and leaf area) was evaluated only using the unfiltered dataset (original foliage-flower dataset). Nitrogen (N), phosphorus (P), and potassium (K) deficient Hoagland’s solution limited petunia growth (**Figure 8A**). The effect of N and K deficiency on petunia growth appeared at week 3. In contrast, growth limitation was observed from week 2 in P deficient petunias. At week 5, the average digital biomass of N, P, and K deficient petunias was 80%, 90%, and 50% smaller than control petunias, respectively (**Figure 8B**). Similarly, the 3D leaf area of N, P, and K deficient petunias was 70%, 86%, and 32% smaller than control petunias, respectively (**Figure 8C**). However, the leaf area decrease in K deficient petunias was not statistically significant.

The average color profile (hue) of petunias was evaluated on the scan made on week 5 after manually removing all flowers. The color profile of control petunia plants irrigated with the complete Hoagland’s solution was 9.02% yellow, 0.06% blue, 1.35% red, and 89.57% green (**Figure 8D**). In the N deficient petunias, the proportion of green foliage decreased to 83.97%, and the proportion of yellow increased to 14.67%. In the P deficient petunias, the proportion of green foliage decreased to 68.63%, and the proportion of yellow, blue, and red foliage increased to 28.42%, 0.35%, and 2.59%, respectively. In K deficient petunias, the proportion of green significantly decreased to 81.83%, and the proportion of yellow increased to 16.59% (**Figure 8D**).

**Figure 9:**
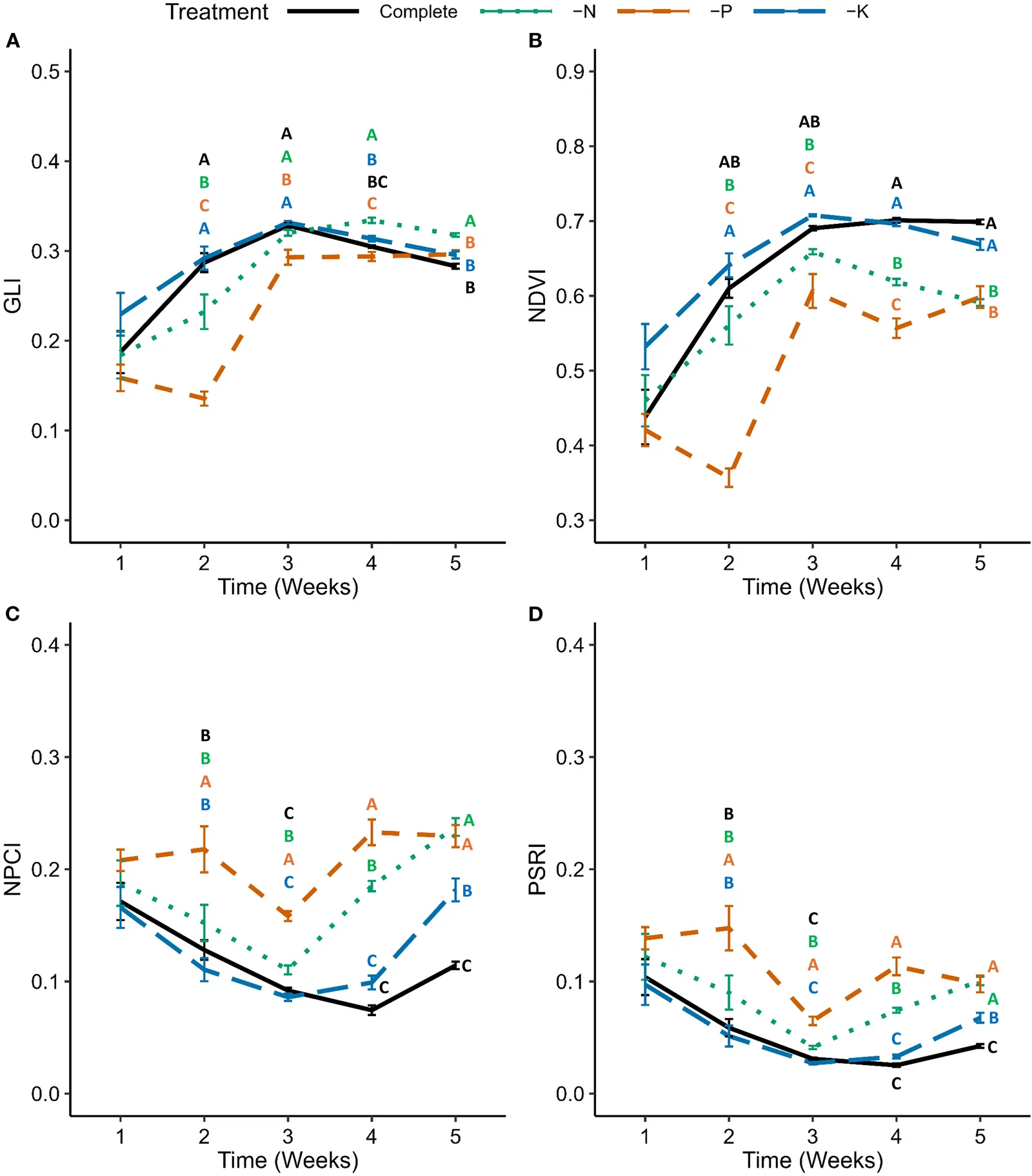
Petunias were grown with modified Hoagland’s solution containing all nutrients (complete, control) or modified Hoagland’s solutions deficient in nitrogen (-N), phosphorus (-P), or potassium (-K) and evaluated weekly for differences in vegetation indices. GLI filtering of the 3D scanned images was used to digitally exclude all flower tissue from the dataset. The indices measured include: GLI [green leaf index, **(A)**], NDVI [normalized difference vegetation index, **(B)**], NPCI [normalized pigment chlorophyll index, **(C)**], and PSRI [plant senescence reflectance index, **(D)**]. For each plot, lines represent the nutrient solution treatments and indicate the mean index for each week, error bars represent the standard error (n = 8), and letters represent significant differences between nutrient solution treatments at each week (p < 0.05).

Because of the influence of flower tissue on vegetation indices, we evaluated petunia foliage health using the GLI filtered data. GLI was only a good indicator of N deficiency (**Figure 9A**), NDVI was a good indicator for N and P deficiency (**Figure 9B**), and NPCI and PSRI were good indicators of N, P, and K deficiency (**Figures 9C, D**). Average vegetative indices for control petunias irrigated with complete Hoagland’s solution ranged from 0.19 to 0.33 for GLI, from 0.44 to 0.70 for NDVI, from 0.07 to 0.17 for NPCI, and from 0.03 to 0.10 for PSRI (**Figure 9**). At week 5, GLI of N deficient petunias was 12% higher than the control petunias (**Figure 9A**). In contrast, the NDVI values of N and P deficient petunias were 15% and 14% lower than the control plants, respectively (**Figure 9B**). In N, P, and K deficient petunias at 5 weeks, NPCI was 108%, 101%, and 59% higher than the control plants, and PSRI was 136%, 130%, and 59% higher than the control petunias (**Figures 9C, D**).

### 3.7 Digital phenotyping of celosia plants grown under nitrogen, phosphorus, and potassium deficiency

**Figure 10:**
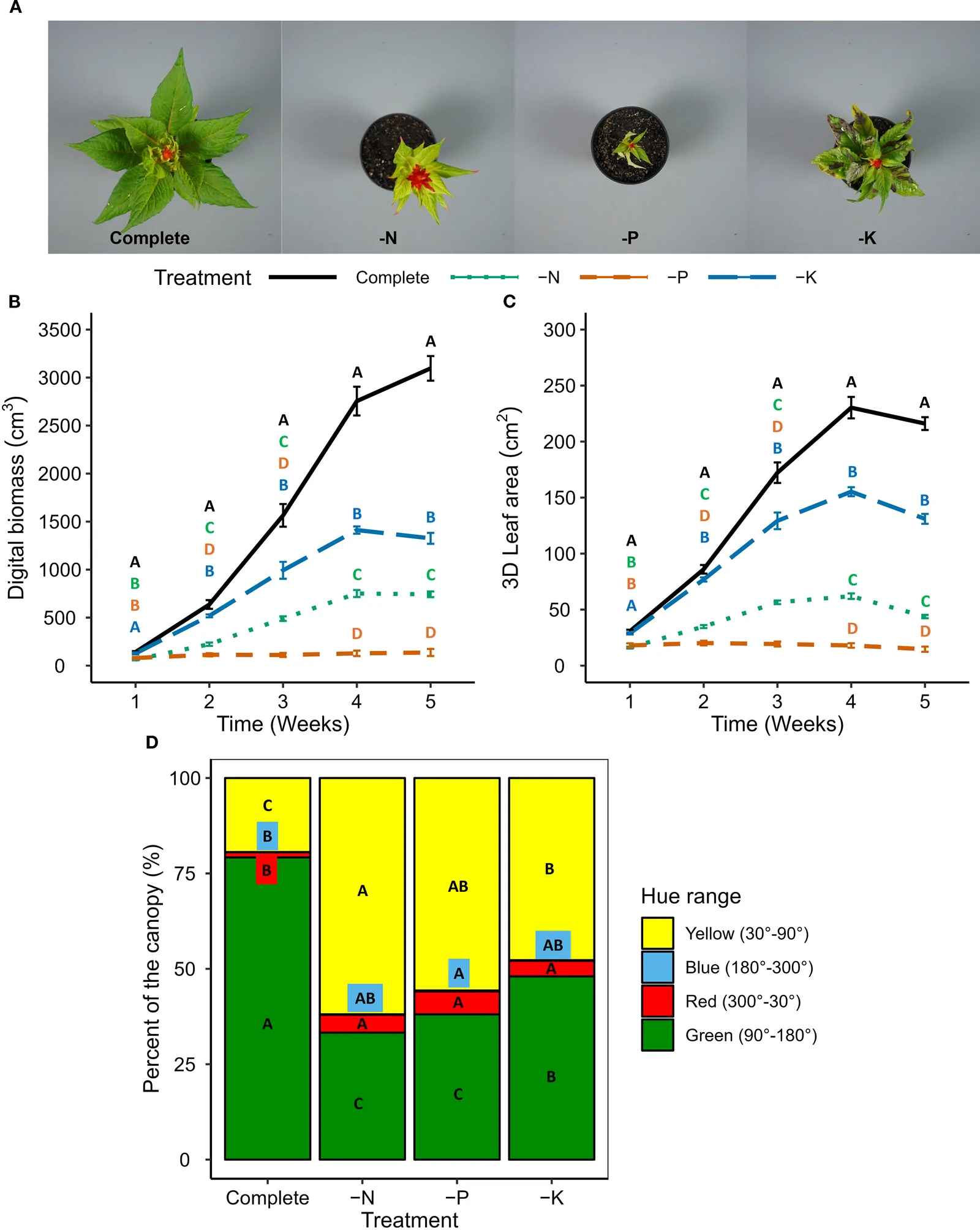
Celosia plants were grown with modified Hoagland’s solution containing all nutrients (complete, control) or modified Hoagland’s solutions deficient in nitrogen (-N), phosphorus (-P), or potassium (-K) and evaluated for morphological differences. Representative celosia five weeks from treatment initiation **(A)**. Digital biomass **(B)** and 3D leaf area **(C)** of celosia irrigated with the different nutrient solutions were evaluated weekly. Data is from unfiltered 3D scanned images. Lines represent the nutrient solution treatments and indicate the mean measured value for each week; error bars represent the standard error (n = 8), and different letters indicate significant differences between nutrient solution treatments at each week (p < 0.05). Plant foliage color was evaluated with spectral reflectance data, where the percentage of the leaf canopy associated with each hue range after five weeks is presented **(D)**. Data is from unfiltered 3D scanned images taken after manually removing all flowers. Letters represent significant differences between treatments for each hue range (n = 8, p < 0.05).

Celosia growth (digital biomass and 3D leaf area) was evaluated only using the unfiltered dataset (original foliage-flower dataset). Nitrogen (N), phosphorus (P), and potassium (K) deficient Hoagland’s solution limited celosia growth (**Figure 10A**). The effect of N and P deficiency on celosia growth appeared at week 1, while growth limitation was observed from week 2 in K deficient celosias. At week 5, the average digital biomass of N, P, and K deficient celosia plants was 76%, 96%, and 57% smaller than the control plants, respectively (**Figure 10B**). Similarly, the 3D leaf area of N, P, and K deficient celosias was 80%, 93%, and 39% smaller than the control plants, respectively (**Figure 10C**).

The average color profile (hue) of celosia foliage was evaluated on the scan made on week 5 after manually removing all flowers. The color profile of control celosia plants irrigated with the complete Hoagland’s solution was 19.42% yellow, 0.05% blue, 1.35% red, and 79.19% green (**Figure 10D**). In the N deficient celosias, the proportion of green foliage decreased to 33.27%, and the proportion of yellow and red increased to 61.92% and 4.66%. In the P deficient celosias, the proportion of green foliage decreased to 38.07%, and the proportion of yellow, blue, and red foliage increased to 55.68%, 0.26%, and 5.99%, respectively. In K deficient celosias, the proportion of green decreased to 48.01%, and the proportion of yellow and red increased to 47.74% and 4.10%, respectively (**Figure 10D**).

**Figure 11:**
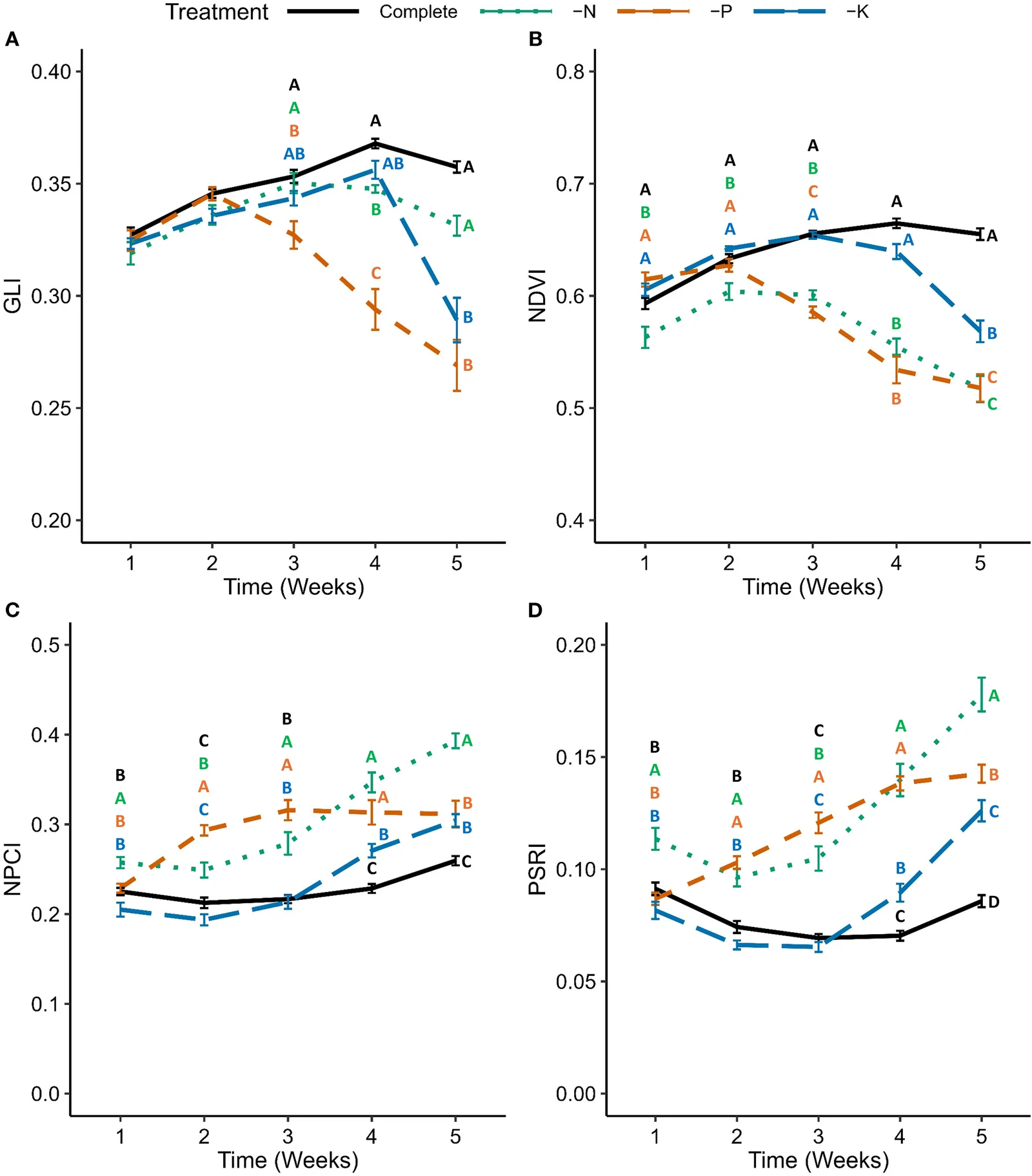
Celosia plants were grown with modified Hoagland’s solution containing all nutrients (complete, control) or modified Hoagland’s solutions deficient in nitrogen (-N), phosphorus (-P), or potassium (-K) and evaluated weekly for differences in vegetation indices. PSRI filtering of the 3D scanned images was used to digitally exclude all flower tissue from the dataset. The indices measured include: GLI [green leaf index, **(A)**], NDVI [normalized difference vegetation index, **(B)**], NPCI [normalized pigment chlorophyll index, **(C)**], and PSRI [plant senescence reflectance index, **(D)**]. For each plot, lines represent the nutrient solution treatments and indicate the mean index for each week, error bars represent the standard error (n = 8), and letters represent significant differences between nutrient solution treatments at each week (p < 0.05).

Vegetation indices (GLI, NDVI, NPCI, and PSRI) for celosia were evaluated with the PSRI filtered dataset that excluded flowers (**Figure 11**). GLI was a good indicator of N and P deficiency (**Figure 11A**). NDVI, NPCI, and PSRI were good indicators of N, P, and K deficiency (**Figures 11B–D**). In celosias fertilized with the complete Hoagland’s solution (control), the average vegetative indices were 0.33 to 0.37 for GLI, 0.59 to 0.66 for NDVI, 0.21 to 0.26 for NPCI, and 0.07 to 0.09 for PSRI (**Figure 11**). Compared to the control plants, P and K deficiency decreased the GLI (**Figure 11A**), and N, P, and K deficiency decreased the NDVI (**Figure 11B**). All deficiency treatments increased NPCI and PSRI in celosia plants (**Figures 11C, D**). By week 5, the GLI values for P and K deficient celosias were 25% and 19% lower than the control plants, respectively. Similarly, the NDVI of N, P, and K deficient celosias was 21%, 21%, and 13% lower than the control celosias, respectively. In contrast, N, P, and K deficient celosias showed a 51%, 20%, and 17% higher NPCI than control plants, respectively. Similarly, N, P, and K deficient solutions resulted in 107%, 66%, and 47% higher NPCI than the complete nutrient solution treatment (control), respectively.

## 4 Discussion

An adequate nutritional management program for ornamental and vegetable bedding plants requires knowledge about nutrient deficiency symptomology. Although there are some general symptoms associated with specific nutritional deficiencies, symptomology is highly species dependent (Gibson et al., 2007). For instance, leaf purpling is a common symptom of P deficiency. However, not all plants purple in response to P deficiency (Hughes and Lev-Yadun, 2015). Moreover, there are some ornamental plants (i.e., coleus) with purple foliage, even when all nutrients are supplied (Whipker et al., 2024). The main objective of this project was to characterize the plant responses to macronutrient deficiency in five species of greenhouse bedding plants using a digital phenotyping platform.

N, P, and K deficiency reduced growth as confirmed by digital biomass and leaf area data. In all species evaluated, the strongest growth reduction was caused by P deficiency, followed by N and then K deficiency. For instance, the shoot digital biomass of P deficient coleus plants was 99% lower than the control plants at the end of the experiment. Across all species, differences in growth were detected the most quickly in P deficient plants, followed by N and K deficient plants (**Figures 2B**, **4B**, **6B**, **8B**, **10B**).

The TraitFinder enhances experimental throughput by increasing the number of plants and the number of parameters evaluated per unit of time. For instance, each scan collects morphological (e.g., digital biomass and leaf area) and spectral information (i.e., the amount of R, G, B, and NIR light reflected by the canopy). The amount of light reflected by the leaves at specific wavelengths can provide insight about the physiological status or health of the plant (Jakomulska et al., 2002). Green and healthy foliage commonly shows high NIR reflectance and low RGB reflectance (high RGB absorbance) (Jakomulska et al., 2002). The spectral information is used to calculate four vegetation indices (GLI, NDVI, NPCI, and PSRI) commonly used to quantitatively evaluate plant heath (Montero et al., 2023).

GLI, NDVI, NPCI, and PSRI have been previously measured in various agronomic crops (Bazhenov et al., 2023), tomato (Sudiro et al., 2022), and some ornamental species (Wang et al., 2012). Sudiro et al. (2022) measured all four vegetation indices in healthy and drought stressed tomato ‘Money Maker’ using a PlantEye 500 (an earlier version of the scanners in the TraitFinder). However, no differences were observed between the vegetation indices of healthy and drought-stressed tomatoes. In ornamentals, NDVI has been used to estimate N status in geranium (*Pelargonium × hortorum*) (Wang et al., 2012). To establish reference values for healthy plants (plants grown with complete nutrient solution), we averaged each vegetation index for each species using data collected from four weeks after transplant to harvest (**Table 3**). Although data from week 1 to week 3 is presented for all species, we did not include data from week 1 to week 3 in the calculation of reference values because of the potential influence of physiological stage on leaf pigments (Sims and Gamon, 2002) and therefore on light reflectance.

**Table 3:** Average vegetation index values for plant species irrigated with a complete Hoagland’s solution.

Parameter	Coleus	Tomato	Marigold	Petunia	Celosia
Period (week)	4 to 5	4 to 6	4 to 6	4 to 5	4 to 5
GLI	0.35 ± 0.002	0.21 ± 0.007	0.30 ± 0.005	0.29 ± 0.003	0.36 ± 0.002
NDVI	0.42 ± 0.005	0.53 ± 0.009	0.74 ± 0.003	0.70 ± 0.002	0.66 ± 0.003
NPCI	0.46 ± 0.005	0.15 ± 0.005	0.09 ± 0.004	0.09 ± 0.006	0.24 ± 0.005
PSRI	0.26 ± 0.005	0.09 ± 0.005	0.03 ± 0.001	0.03 ± 0.002	0.08 ± 0.003

Values represent the mean ± standard error of measurements taken over the specified period.

GLI values can range from −1.0 to 1.0, and zero can serve as a general threshold to distinguish between green vegetation and non-living objects like soil or growing substrate (Louhaichi et al., 2001). GLI is positively correlated with leaf and plant N content (Kior et al., 2024), but it is negatively correlated with leaf pigment concentrations (Blackmer et al., 1994; Clemente et al., 2023). In healthy tomato ‘Money Maker’, GLI is about 0.25 and does not change in response to drought stress (Sudiro et al., 2022). In contrast, GLI was shown to decrease from about 0.20 to 0.075 in wheat (*Triticum aestivum* L.) cultivars exposed to 30 days of drought (Moritz et al., 2024). From week four until harvest, the average GLI of healthy coleus, tomato, marigold, petunia, and celosia (fertilized with complete Hoagland’s) was 0.35 ± 0.002, 0.21 ± 0.007, 0.30 ± 0.005, 0.29 ± 0.003, and 0.36 ± 0.002, respectively (**Table 3**).

NDVI values associated with healthy plants range from 0.5 to 1.0 (Jakomulska et al., 2002). NDVI values decrease with the onset of senescence (Hatfield and Prueger, 2010) or in response to abiotic stress (Govind et al., 2005; Tafesse et al., 2022). Petunias fertilized with 50 mg L^-1^ N 15N-2.2P-12.5K-2.9Ca-1.2Mg (half the recommended rate) have an NDVI of ~ 0.7 (Quijia Pillajo et al., 2023). Geraniums grown under optimal fertilization have an NDVI of ~ 0.8 (Wang et al., 2012). The NDVI of healthy tomato ‘Money Maker’ is about 0.60 and does not change in response to drought stress (Sudiro et al., 2022). In healthy coleus, tomato, marigold, petunia, and celosia we observed an average NDVI of 0.42 ± 0.005, 0.53 ± 0.009, 0.74 ± 0.003, 0.70 ± 0.002, and 0.66 ± 0.003, respectively (**Table 3**). In geranium, NDVI values decrease in response to N limitation (Wang et al., 2012). Similarly, we observed that NDVI values decreased in response to N, but also P and K deficiency.

NPCI values associated with healthy plants are from −0.1 to 0.2 (Bazhenov et al., 2023). NPCI values correlate with chlorophyll content and increase upon chlorophyll degradation during senescence (Hatfield and Prueger, 2010). NPCI values also increase in response to environmental stress (i.e., heat and nitrogen stress) (Govind et al., 2005; Tafesse et al., 2022) or pathogen attack (i.e., pepper mosaic virus) (Kshirsagar et al., 2019). By the end of each experiment, we observed an average NPCI of 0.46 ± 0.005, 0.15 ± 0.005, 0.09 ± 0.004, 0.09 ± 0.006, and 0.24 ± 0.005 in healthy coleus, tomato, marigold, petunia, and celosia plants (after flowers and buds were removed from flowering plants), respectively (**Table 3**). As previously reported, we also observed that NPCI values increased in response to nutrient deficiency. However, the response depended on the plant species and nutrient tested. N deficiency increased NPCI in all species tested, P deficiency increased NPCI in marigold, petunia, and celosia, and K deficiency increased NPCI in petunia and celosia. In contrast, the NPCI of tomato decreased in response to P deficiency compared to the control tomato plants (**Figure 5C**). A similar response has been observed in tomatoes grown under low fertility (50 mg/L N 15-5-15) and treated with different doses of humic substances. While biomass of tomato plants treated with humic substances under low fertility were higher than those not treated with humic substances, these plants also had higher NPCI (Quijia Pillajo et al., 2024). Thus, tomato plants seem to produce a specific spectral profile under abiotic stress that is different than the ornamental species we have tested.

A PSRI associated with healthy plants ranges from − 0.1 to 0.2 (Lowe et al., 2017; Bazhenov et al., 2023), and values increase upon senescence (Hatfield and Prueger, 2010). By the end of each experiment, we observed an average PSRI of 0.26 ± 0.005, 0.09 ± 0.005, 0.03 ± 0.001, 0.03 ± 0.002, and 0.08 ± 0.003, in healthy coleus, tomato, marigold, petunia, and celosia plants (after flowers and buds were removed), respectively (**Table 3**). In marigold, NPCI and PSRI values increase in response to P limitation (Quijia-Pillajo et al., 2025). As previously reported, we also observed that PSRI values increased in response to nutrient deficiency. However, the response depended on the plant species and nutrient tested. N deficiency increased PSRI in all species tested, P deficiency increased PSRI only in marigold, petunia, and celosia, and K deficiency increased PSRI only in petunia and celosia. Similar to what we observed for tomato NPCI, instead of an increase, the PSRI of tomato decreased in response to P deficiency and was lower than the control (**Figure 5D**).

Leaf pigment (i.e., chlorophyll, anthocyanins, and carotenoids) content influences the reflectance of visible light (Slaton et al., 2001; Sims and Gamon, 2002; Ollinger, 2011), and leaf yellowing or chlorosis is a N deficiency symptom resulting from the degradation of chlorophylls (Gaude et al., 2007; de Bang et al., 2021). Leaf purpling is a P deficiency symptom resulting from the accumulation of anthocyanins that function as antioxidants (Havlin et al., 2014; de Bang et al., 2021). K deficiency increases sensitivity to light levels and causes photo-oxidative damage to chloroplasts, which causes leaf tissue to become chlorotic and necrotic. In K-deficient plants, the generation of excess reactive oxygen species leads to oxidative degradation of chlorophyll and membranes (Cakmak, 2005; de Bang et al., 2021). Thus, changes in light reflectance can be used to quantify N, P, and K deficiency stress. For instance, P deficient soybean showed an increase in reflectance of the green and yellow portion of the spectrum and a shift in the red edge position (Milton et al., 1991). Accordingly, anthocyanins are reported to absorb light in the green and yellow portion of the spectrum (Karageorgou and Manetas, 2006). Physiological stage can also influence leaf pigments; for instance, the early vegetative stage is also characterized by leaves with high anthocyanin content and low photosynthetic activity (Sims and Gamon, 2002).

Similar to other visual symptoms, the spectral signature associated with macronutrient deficiency is species dependent (Shi et al., 2012). Over time we observed a common trend. Compared to the control plants (complete nutrients), GLI and NDVI decreased and NPCI and PSRI increased in response to macronutrient deficiencies. Similarly, GLI and NDVI decreased and NPCI and PSRI increased due to a potential phytotoxicity in tomato ‘Bush Beefsteak’ treated with a fulvic acid-based biostimulant product (Quijia Pillajo et al., 2024). However, we found some exceptions. For instance, in tomato ‘Early Girl Plus’ NDVI increased and NPCI and PSRI decreased under P deficiency. Similar results were observed in wheat and rye, where the NPCI and PSRI decreased in response to P deficiency (Bazhenov et al., 2023). The divergent response to P deficiency observed in tomato may be attributed to a restricted leaf development that in turn may increase chlorophyll concentration per unit area leading to stronger RED-light absorption (Hecht‐Buchholz, 1967). In contrast to species like marigold, anthocyanin accumulation was primarily observed on the abaxial leaf surface. Because of this location, anthocyanin may have limited influence on reflectance profiles measured from the adaxial surface (Hughes et al., 2008), as is typical in top-down imaging systems like TraitFinder. Another exception to our general observation was seen in petunia, where N deficiency resulted in a 12% higher GLI than control petunias (**Figure 9A**). However, based on the observed petunia NDVI, NPCI, and PSRI response to N deficiency, we believe GLI is not a reliable indicator in this case. Accordingly, it has been shown that RGB-based indices are less sensitive than NIR-based indices like NDVI (Yeom et al., 2019).

Although we observed a common response of vegetation indices to macronutrient deficiency in the plants evaluated in this experiment, we also observed some exceptions. These results underscore the importance of accounting for the diversity of spectral signatures when evaluating responses to nutrient deficiency or other biotic or abiotic stresses. The pigments and internal structure of petals create distinct spectral signatures, making it difficult to interpret reflectance spectral data from flowering plants (Ollinger, 2011; Van Der Kooi et al., 2016; Quijia Pillajo et al., 2023). We also found that flowers can significantly influence the calculation of vegetation indices. To ensure accuracy, flowers should be excluded from the analysis. This is possible with advanced digital phenotyping systems. Such systems not only enhance experimental throughput but also improve the accuracy and precision of plant evaluation.

Digital biomass and leaf areas are reliable indicators of plant growth, and vegetation indices effectively reflect canopy physiological status. However, caution should be taken when these variables are derived from scans in which flowers are digitally removed. The TraitFinder does not capture areas of the canopy shaded or occluded by the flowers. Therefore, any underlying canopy area is excluded after filtering because those data points were not scanned. This limitation is particularly important for heavy-flowering species where occlusion might be more severe than was observed in this study. To minimize bias associated with data loss during filtering in heavy-flowering species, manual flower removal prior to scanning is recommended, or plants should be evaluated before reaching full blooming stage.

## Data Availability

The original contributions presented in the study are included in the article/**Supplementary Material**. Further inquiries can be directed to the corresponding author.
