# Correction: Macronutrient deficiency reduces growth and influences vegetation indices of greenhouse grown ornamental and vegetable plants as measured by the TraitFinder digital phenotyping system

**DOI:** 10.3389/fpls.2026.1945522

**Published:** 2026-08-13

**Authors:** Juan Quijia-Pillajo, Janhavi Maurya, Laura J. Chapin, Michelle L. Jones

**Affiliations:** Department of Horticulture and Crop Science, The Ohio State University, Wooster, OH, United States

**Keywords:** celosia, coleus, marigold, nutrient stress, petunia, PlantEye F600, soilless substrate, tomato

The hue ranges for the green and blue color categories were incorrectly reported.

A correction has been made to section **2 Materials and methods**, subsection *2.3 Digital phenotyping*, Paragraph 2. It stated: “Using HortControl, we calculated the proportion of the canopy falling into four different hue ranges representing red (300° to 30°), yellow (30° to 90°), green (90° to 125°), and blue (125° to 180°) colors”. The correct sentence is:

“Using HortControl, we calculated the proportion of the canopy falling into four different hue ranges representing red (300° to 30°), yellow (30° to 90°), green (90° to 180°), and blue (180° to 300°) colors.”

The inequality symbol for the saturation threshold in marigold was incorrectly reported. Verbiage was added to clarify the filtering.

A correction has been made to section **3 Results**, subsection *3.2 Digital phenotyping of flowering ornamentals*, Paragraph 1. It stated: “The selected filtering parameters were species dependent: saturation **<** 70 for marigold, GLI < 0 for petunia, and PSRI > 0.3 for celosia”. The correct sentence is:

“The selected filtering parameters to remove flowers were species dependent: saturation **>** 70 for marigold, GLI < 0 for petunia, and PSRI > 0.3 for celosia.”

The comparative relationship of the NPCI and PSRI responses were incorrectly reported in one sentence.

A correction has been made to section **3 Results**, subsection *3.4 Digital phenotyping of tomato plants grown under nitrogen, phosphorus, and potassium deficiency,* Paragraph 4. It stated: “P deficient tomato plants showed lower GLI from week 2 to 6, and greater NPCI and PSRI than control plants from week 4 to 6 **(Figures 5A, D)**”. The correct sentence is:

“P deficient tomato plants showed lower GLI from week 2 to 6, and lower NPCI and PSRI than control plants from week 4 to 6 (Figures 5A, C, D).”

The word “manually” was included in error in two sentences in the **Discussion.**

A correction has been made to section **4 Discussion**, Paragraph 7. It stated: “By the end of each experiment, we observed an average NPCI of 0.46 ± 0.005, 0.15 ± 0.005, 0.09 ± 0.004, 0.09 ± 0.006, and 0.24 ± 0.005 in healthy coleus, tomato, marigold, petunia, and celosia plants (after flowers and buds were manually removed from flowering plants), respectively (Table 3)”. The correct sentence is:

“By the end of each experiment, we observed an average NPCI of 0.46 ± 0.005, 0.15 ± 0.005, 0.09 ± 0.004, 0.09 ± 0.006, and 0.24 ± 0.005 in healthy coleus, tomato, marigold, petunia, and celosia plants (after flowers and buds were removed from flowering plants), respectively (Table 3).”

A correction has been made to section **4 Discussion**, Paragraph 8. It stated: “By the end of each experiment, we observed an average PSRI of 0.26 ± 0.005, 0.09 ± 0.005, 0.03 ± 0.001, 0.03 ± 0.002, and 0.08 ± 0.003, in healthy coleus, tomato, marigold, petunia, and celosia plants (after flowers and buds were manually removed), respectively **(Table 3)**”. The corrected sentence is:

“By the end of each experiment, we observed an average PSRI of 0.26 ± 0.005, 0.09 ± 0.005, 0.03 ± 0.001, 0.03 ± 0.002, and 0.08 ± 0.003, in healthy coleus, tomato, marigold, petunia, and celosia plants (after flowers and buds were removed), respectively (Table 3).”

The original version of this article has been updated.

