# Supplementary material for: Macronutrient deficiency reduces growth and influences vegetation indices of greenhouse grown ornamental and vegetable plants as measured by the TraitFinder digital phenotyping system
Source: Front Plant Sci. 2026 May 12;17:1702429. doi: 10.3389/fpls.2026.1702429 (PMC13201125; doi:10.3389/fpls.2026.1702429)
Supplement: Supplementary file 1 [file DataSheet1.zip › Supplementary file 1.pdf]

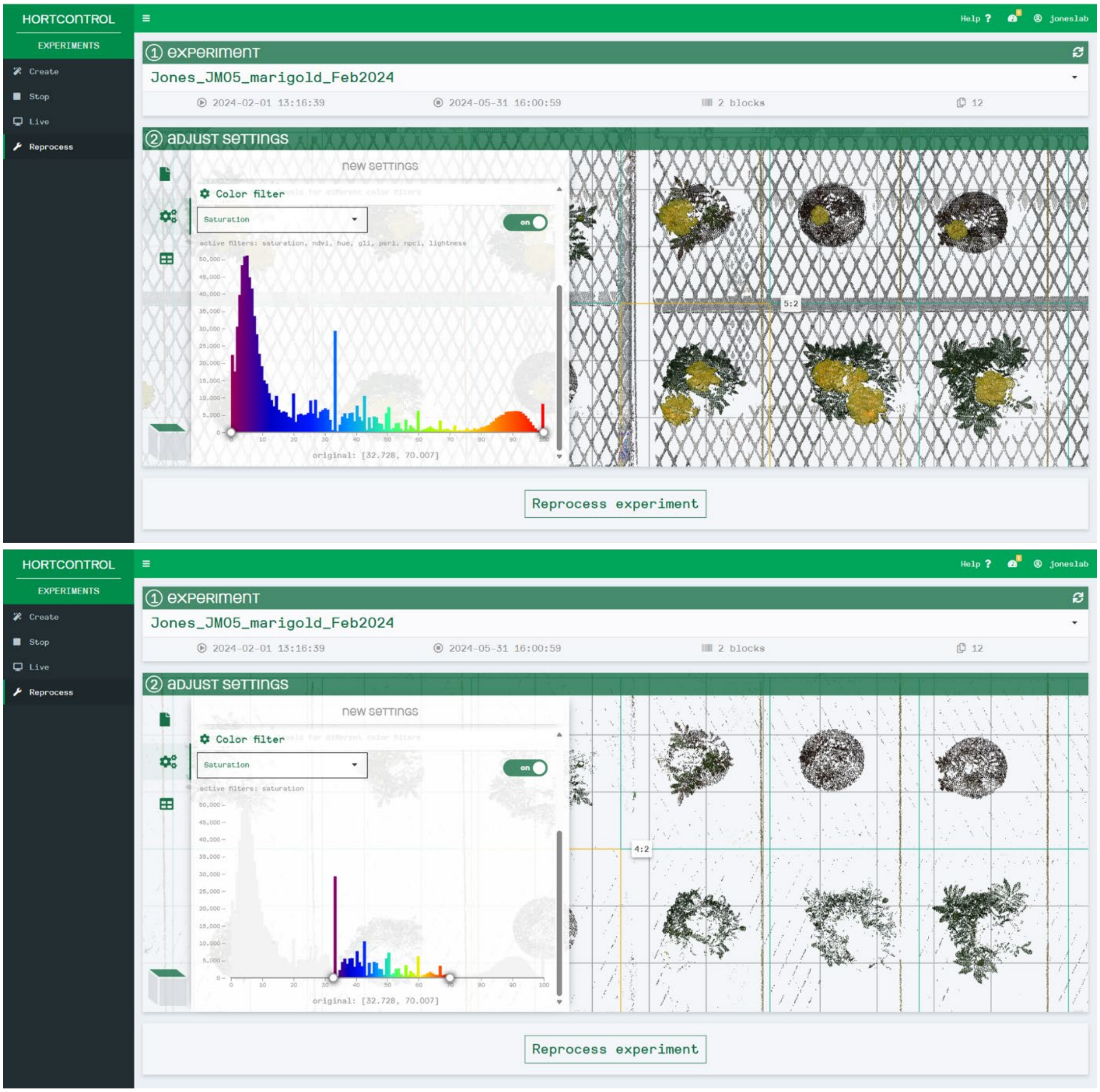

Figure S1 Saturation data histogram and 3D view of French marigolds. The top panel shows scans with no saturation filtering applied. The bottom panel shows scans after applying a saturation threshold, retaining values between 70 and 100.

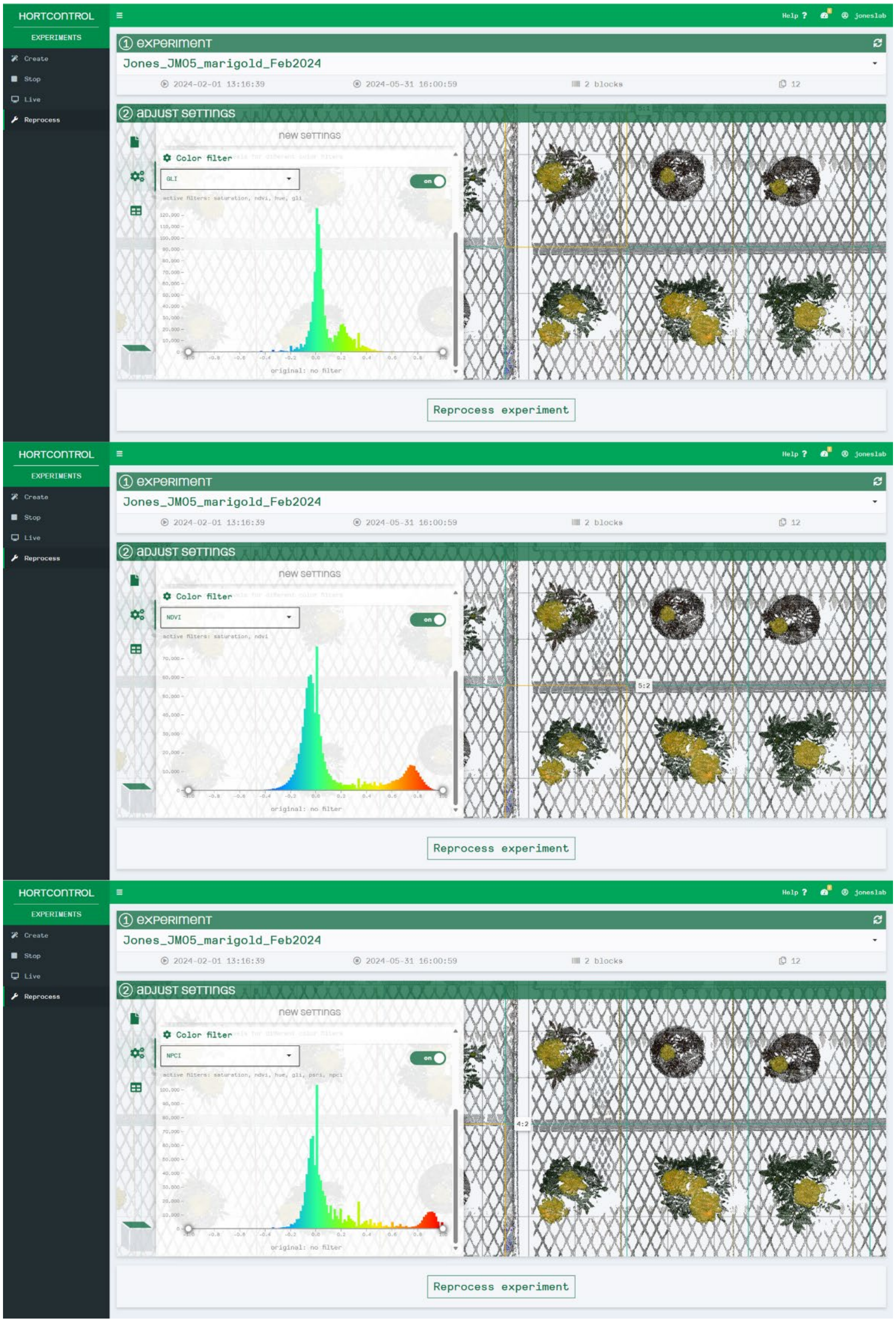

Figure S2 Green leaf index (GLI) data histogram and 3D view of French marigolds (Top). Normalized difference vegetation index (NDVI) data histogram and 3D view of French marigolds (Middle). Normalized pigment chlorophyll ratio index (NPCI) data histogram and 3D view of French marigolds (Bottom).

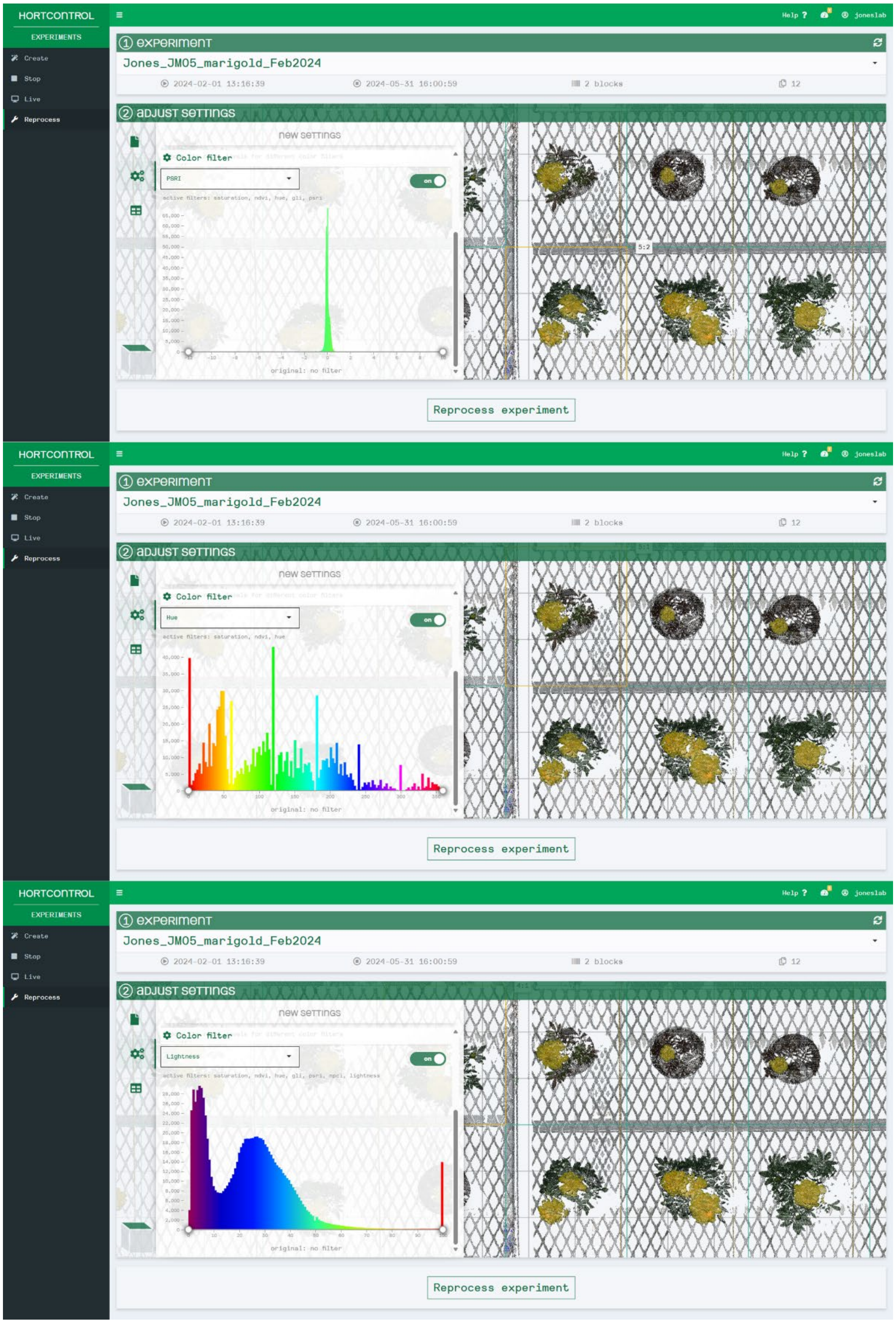

Figure S3 Plant senescence reflectance index (PSRI) data histogram and 3D view of French marigolds (Top). Hue data histogram and 3D view of French marigolds (Middle). Lightness data histogram and 3D view of French marigolds (Bottom).

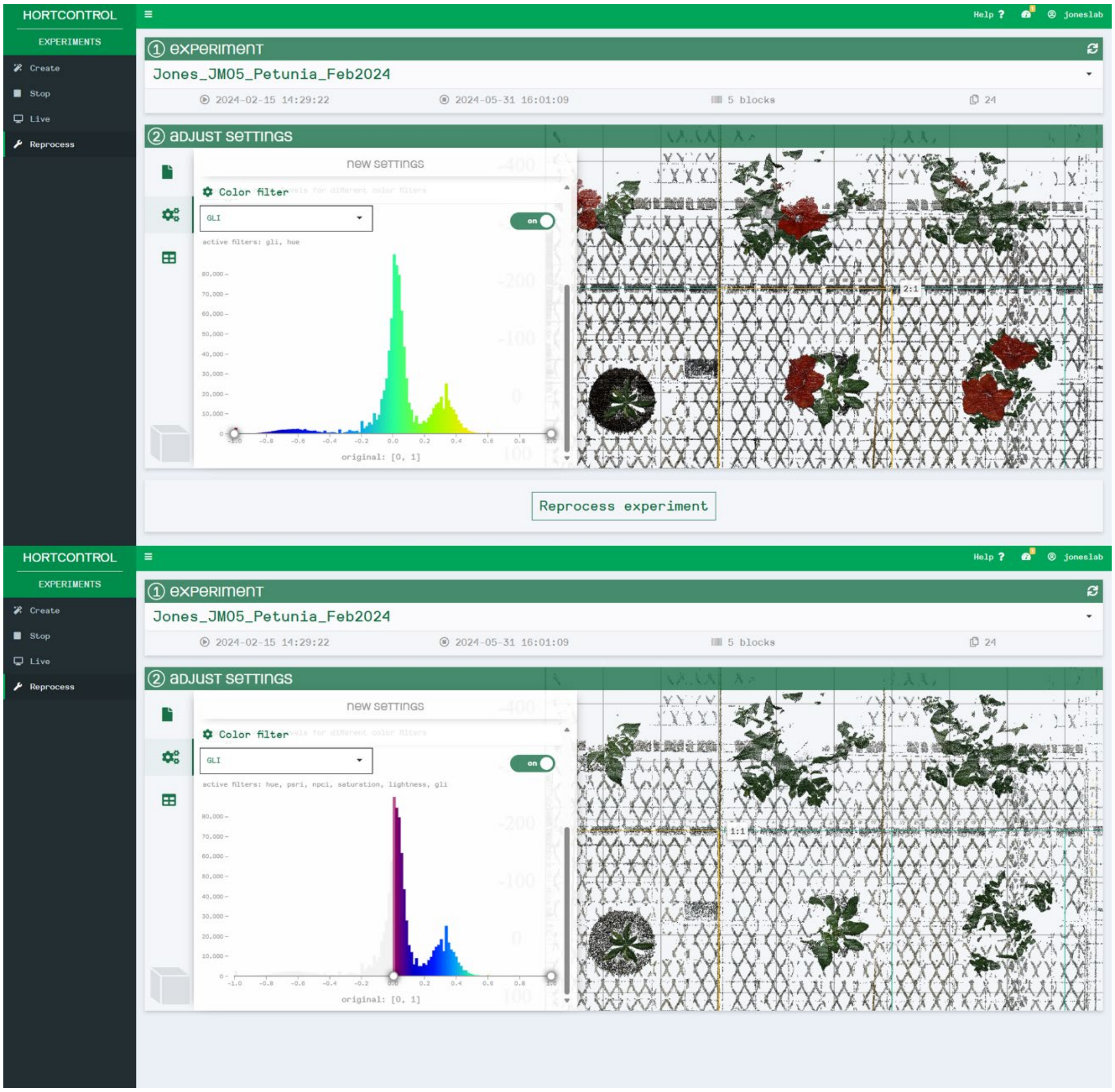

Figure S4 Green leaf index (GLI) data histogram and 3D view of petunia. The top panel shows scans with no GLI filtering applied. The bottom panel shows scans after applying a GLI threshold, retaining values > 0.

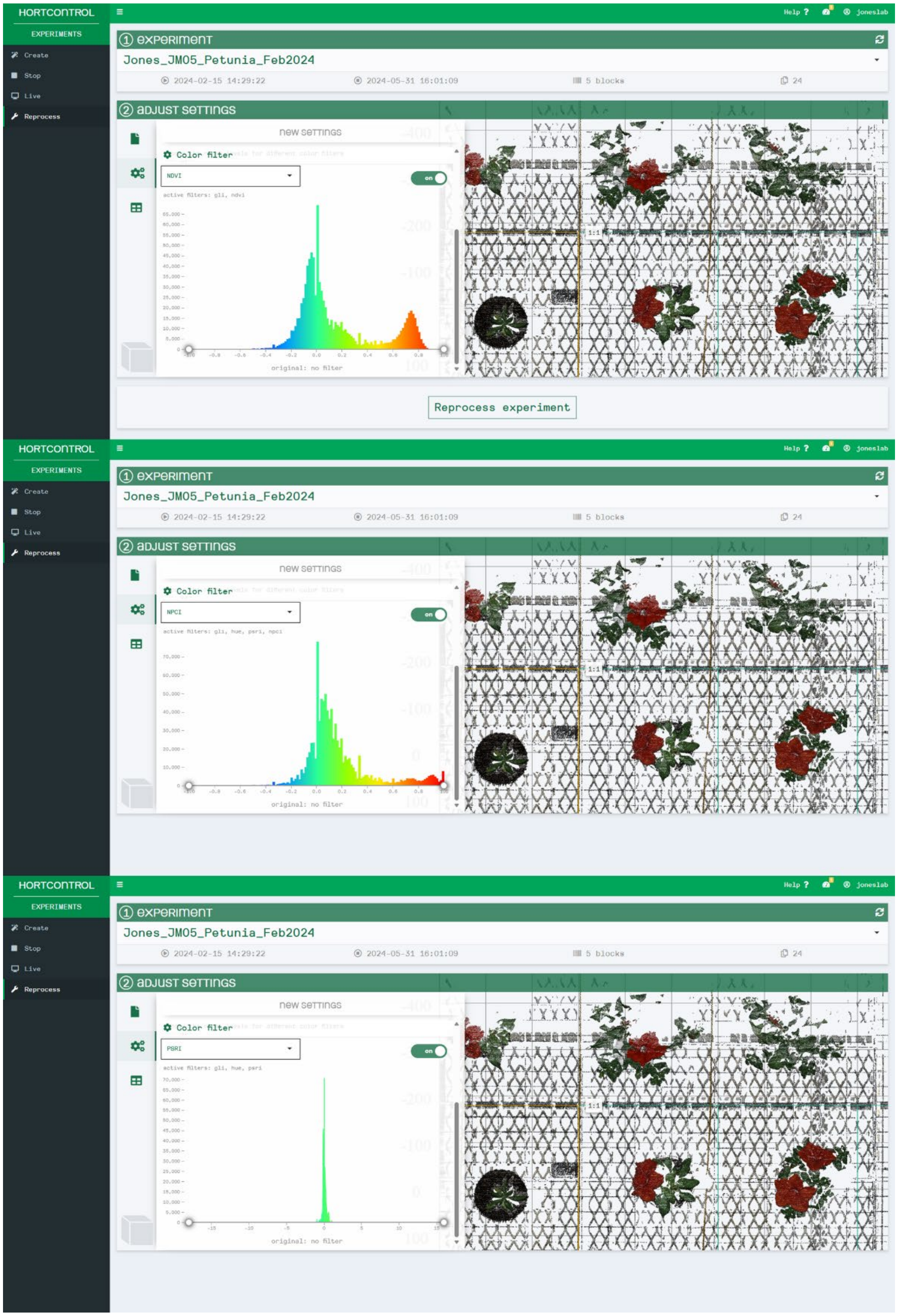

Figure S5 Normalized difference vegetation index (NDVI) data histogram and 3D view of petunia (Top). Normalized pigment chlorophyll ratio index (NPCI) data histogram and 3D view of petunia (Middle). Plant senescence reflectance index (PSRI) data histogram and 3D view of petunia (Bottom).

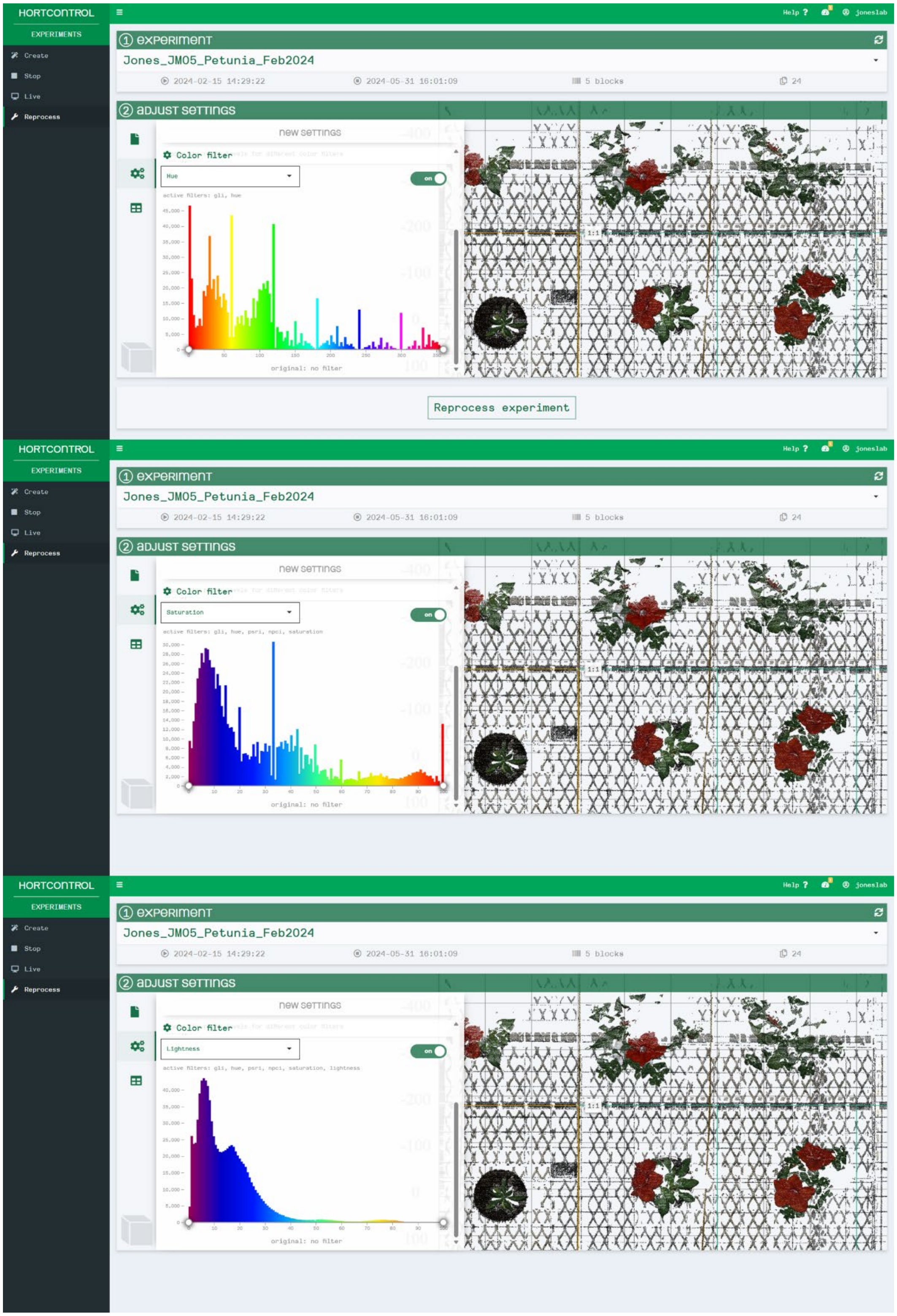

Figure S6 Hue data histogram and 3D view of petunia (Top). Saturation data histogram and 3D view of petunia (Middle). Lightness data histogram and 3D view of petunia (Bottom).

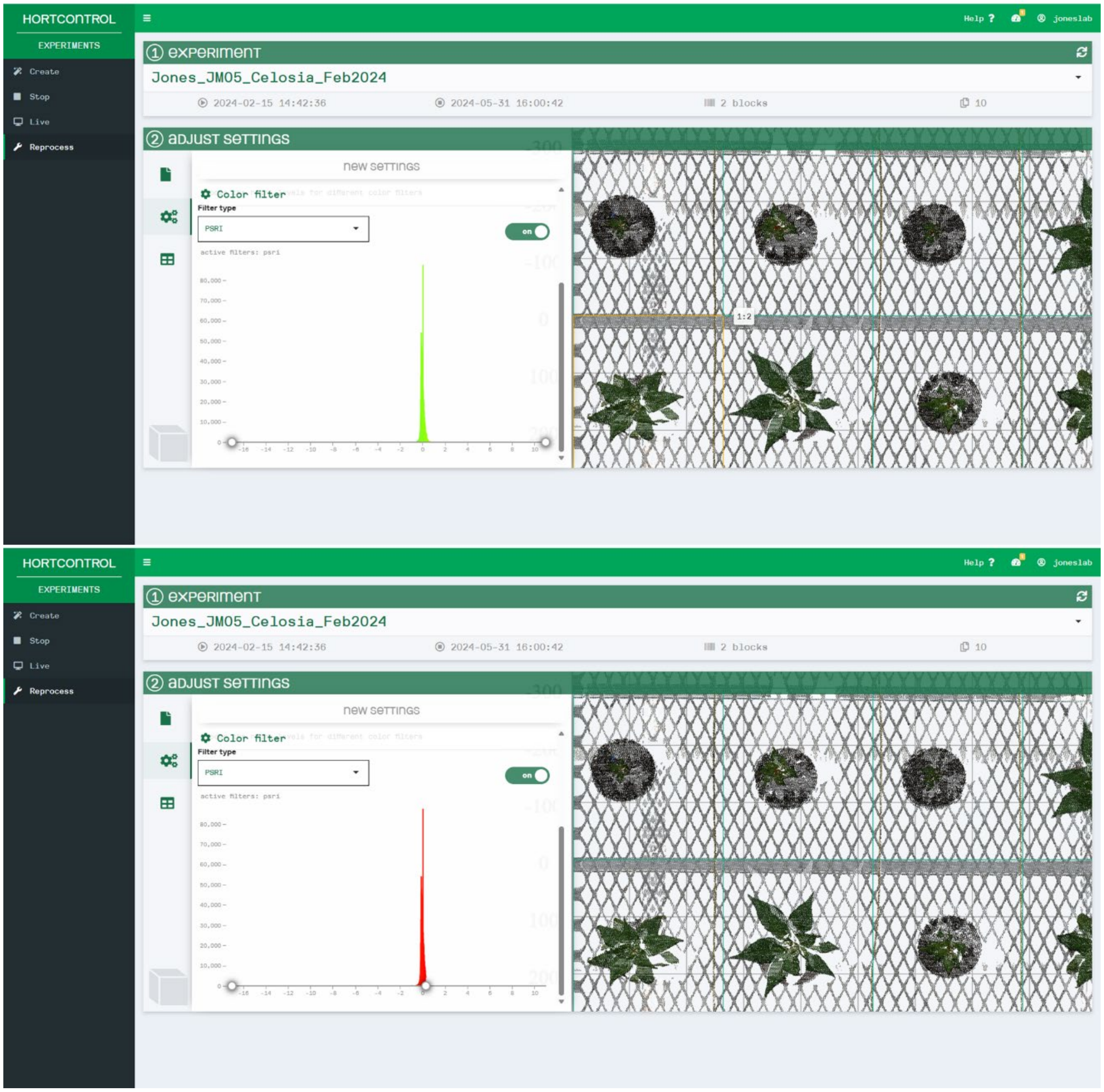

Figure S7 Plant senescence reflectance index (PSRI) data histogram and 3D view of celosia. The top panel shows scans with no PSRI filtering applied. The bottom panel shows scans after applying a PSRI threshold, retaining values < 0.3.
